# Supplementary material for: XBB.1.5 monovalent mRNA vaccine booster elicits robust neutralizing antibodies against XBB subvariants and JN.1
Source: Cell Host Microbe. 2024 Mar 13;32(3):315–321.e3. doi: 10.1016/j.chom.2024.01.014 (PMC10948033; doi:10.1016/j.chom.2024.01.014)
Supplement: Document S1. Figures S1–S4 and Tables S1 and S2 [file mmc1.pdf]

Cell Host & Microbe, Volume *32*

Supplemental information

XBB.1.5 monovalent mRNA vaccine booster elicits robust neutralizing antibodies against XBB subvariants and JN.1

Qian Wang, Yicheng Guo, Anthony Bowen, Ian A. Mellis, Riccardo Valdez, Carmen Gherasim, Aubree Gordon, Lihong Liu, and David D. Ho

Table S1. Summarized participant information, related to Figure 2. Demographic, vaccine, and sera collection information is summarized for each cohort. Listed values represent the mean and range (age and sera collection variables) or number and percentage (vaccine type and sex variables).

Clinical information		All participants		XBB.1.5 MV		XBB infx		Prior Infx + XBB MV			
								Pre-XBB infx + XBB.1.5 MV		XBB infx + XBB.1.5 MV	
		No. or Mean	% or (range)	No. or Mean	% or (range)	No. or Mean	% or (range)	No. or Mean	% or (range)	No. or Mean	% or (range)
	**Total case**	60	-	16	-	19	-	15	-	10	-
	**Female**	47	78.3%	11	68.8%	16	84.2%	12	80.0%	8	80.0%
	**Male**	13	21.7%	5	31.3%	3	15.8%	3	20.0%	2	20.0%
	**Age**	49.7	(30,77)	51.8	(36,65)	48.6	(33,77)	47.8	(35,67)	51.1	(30,62)
**WT monovalent Dose 1 and 2**	Pfizer	53	88.3%	14	87.5%	17	89.5%	14	93.3%	8	80.0%
	Moderna	6	10.0%	2	12.5%	2	10.5%	1	6.7%	1	10.0%
	Janssen	1	1.7%	-	-	-	-	-	-	1	10.0%
**WT monovalent Dose 3**	Pfizer	51	85.0%	14	87.5%	16	84.2%	14	93.3%	7	70.0%
	Moderna	9	15.0%	2	12.5%	3	15.8%	1	6.7%	3	30.0%
**WT monovalent Dose 4**	Pfizer	18	30.0%	5	31.3%	6	31.6%	3	20.0%	4	40.0%
	Moderna	8	13.3%	4	25.0%	2	10.5%	-	-	2	20.0%
	None	34	56.7%	7	43.8%	11	57.9%	12	80.0%	4	40.0%
**BA.5 bivalent booster**	Pfizer	38	63.3%	9	56.3%	16	84.2%	8	53.3%	5	50.0%
	Moderna	22	36.7%	7	43.8%	3	15.8%	7	46.7%	5	50.0%
**XBB.1.5 monovalent booster**	Pfizer	20	33.3%	8	50.0%	-	-	7	46.7%	5	50.0%
	Moderna	21	35.0%	8	50.0%	-	-	8	53.3%	5	50.0%
	None	19	31.7%	-	-	19	100.0%	-	-	-	-
	**Sera Days Pre XBB**	26.5	(1,74)	19.8	(1,74)	30.8	(3,69)	28.1	(2,69)	26.8	(7,55)
	**Sera Days Post XBB**	26.4	(20,34)	26.0	(21,32)	27.8	(22,30)	25.9	(20,34)	24.9	(21,30)

Table S2. Participant details, related to Figure 2. Details are listed for each participant including demographic, vaccine, infection, and sera collection information.

Sample ID	Age	Gender	Infection period	Vaccine type						Days pre/post vaccine/infection		Intervals between 1st dose to					
				WT monovalent vaccine				BA.5 bivalent booster	XBB.1.5 Monovalent booster								
				dose 1	dose 2	dose 3	dose 4			pre sample	post sample	2nd dose	3rd dose	4th dose	BA.5 bivalent booster	Omicron infection	XBB.1.5 Monovalent booster
XBB.1.5 MV (n=16)																	
1	62	Female	-	Pfizer	Pfizer	Pfizer	Pfizer	Pfizer	Moderna	21	27	22	268	491	657	-	993
2	59	Male	-	Pfizer	Pfizer	Pfizer	Pfizer	Pfizer	Pfizer	6	23	21	269	491	622	-	1001
3	65	Female	-	Pfizer	Pfizer	Pfizer	Pfizer	Pfizer	Pfizer	24	28	22	266	503	637	-	990
4	59	Female	-	Pfizer	Pfizer	Pfizer	-	Pfizer	Pfizer	9	28	21	289	-	624	-	992
5	55	Female	-	Pfizer	Pfizer	Pfizer	Moderna	Moderna	Moderna	32	25	21	272	472	637	-	995
6	38	Male	-	Moderna	Moderna	Moderna	-	Moderna	Pfizer	22	27	28	235	-	539	-	914
7	64	Female	-	Pfizer	Pfizer	Pfizer	Pfizer	Pfizer	Pfizer	16	32	21	270	455	606	-	976
8	55	Female	-	Pfizer	Pfizer	Pfizer	Moderna	Moderna	Moderna	23	26	21	291	487	648	-	1005
9	56	Female	-	Pfizer	Pfizer	Pfizer	-	Pfizer	Pfizer	26	21	21	377	-	634	-	993
10	40	Male	-	Pfizer	Pfizer	Pfizer	-	Moderna	Moderna	3	22	21	268	-	584	-	969
11	50	Male	-	Pfizer	Pfizer	Pfizer	Moderna	Pfizer	Moderna	8	31	21	235	444	621	-	996
12	54	Female	-	Pfizer	Pfizer	Pfizer	Moderna	Pfizer	Moderna	1	22	21	329	490	623	-	990
13	38	Female	-	Pfizer	Pfizer	Pfizer	-	Moderna	Pfizer	74	30	21	276	-	619	-	985
14	56	Female	-	Pfizer	Pfizer	Pfizer	Pfizer	Pfizer	Moderna	10	22	21	287	507	654	-	1016
15	36	Male	-	Moderna	Moderna	Moderna	-	Moderna	Moderna	26	26	27	232	-	526	-	905
16	42	Female	-	Pfizer	Pfizer	Pfizer	-	Moderna	Pfizer	16	26	21	239	-	520	-	910
XBB infx (n=19)																	
1	33	Female	2023.02	Pfizer	Pfizer	Pfizer		Pfizer	-	5	30	21	334	-	643	151	-
2	38	Female	2023.08	Pfizer	Pfizer	Pfizer	-	Pfizer	-	53	27	24	294	-	666	307	-
3	59	Female	2023.08	Pfizer	Pfizer	Pfizer	Pfizer	Pfizer	-	37	26	21	295	472	660	280	-
4	44	Male	2023.05	Pfizer	Pfizer	Pfizer	Pfizer	Moderna	-	69	30	21	243	447	621	260	-
5	54	Female	2023.09	Pfizer	Pfizer	Pfizer	Pfizer	Pfizer	-	37	28	21	222	421	602	306	-
6	77	Male	2023.09	Pfizer	Pfizer	Pfizer	Pfizer	Pfizer	-	48	22	21	289	470	615	365	-
7	38	Female	2023.03	Pfizer	Pfizer	Moderna	-	Pfizer	-	14	28	21	278	-	613	99	-
8	59	Female	2023.05	Pfizer	Pfizer	Pfizer	Pfizer	Pfizer	-	24	30	25	281	469	613	249	-
9	41	Female	2023.02	Pfizer	Pfizer	Pfizer	-	Pfizer	-	24	29	21	289	-	659	108	-
10	38	Female	2023.08	Pfizer	Pfizer	Pfizer	-	Pfizer	-	3	30	21	291	-	660	305	-
11	42	Male	2023.04	Pfizer	Pfizer	Pfizer	-	Moderna	-	24	28	21	269	-	615	229	-
12	38	Female	2023.08	Pfizer	Pfizer	Pfizer	-	Pfizer	-	16	24	21	304	-	644	325	-
13	56	Female	2023.09	Pfizer	Pfizer	Pfizer	Pfizer	Pfizer	-	54	29	21	290	492	645	342	-
14	53	Female	2023.02	Pfizer	Pfizer	Pfizer	-	Pfizer	-	16	27	22	320	-	645	140	-
15	40	Female	2023.05	Pfizer	Pfizer	Pfizer	-	Pfizer	-	21	28	21	279	-	658	194	-
16	49	Female	2023.07	Pfizer	Pfizer	Pfizer	-	Moderna	-	63	30	22	328	-	641	264	-
17	59	Female	2023.04	Moderna	Moderna	Moderna	Moderna	Moderna	-	25	24	28	268	505	584	174	-
18	51	Female	2023.05	Moderna	Moderna	Moderna	Moderna	Moderna	-	39	29	28	253	441	554	228	-
19	54	Female	2023.08	Pfizer	Pfizer	Pfizer		Pfizer	-	13	29	23	284	-	632	330	-
Omicron infex + XBB.1.5 MV (n=25)																	
subgroup 1: pre-XBB Omicron infx + XBB.1.5 MV (n=15)																	
1	41	Female	2022.09	Moderna	Moderna	Moderna	-	Pfizer	Moderna	57	25	28	330	-	715	624	1009
2	61	Female	2022.04	Pfizer	Pfizer	Pfizer	-	Moderna	Moderna	22	34	22	302	-	649	487	1006
3	53	Female	2022.04	Pfizer	Pfizer	Pfizer	-	Pfizer	Moderna	35	25	21	275	-	626	471	999
4	49	Male	2022.01	Pfizer	Pfizer	Pfizer	-	Pfizer	Pfizer	12	22	21	265	-	691	320	956
5	67	Female	2022.07	Pfizer	Pfizer	Pfizer	Pfizer	Moderna	Moderna	42	29	21	271	473	690	569	992
6	52	Female	2022.04	Pfizer	Pfizer	Pfizer	Pfizer	Pfizer	Pfizer	27	25	21	266	436	599	460	973
7	48	Female	2022.01	Pfizer	Pfizer	Pfizer	-	Moderna	Pfizer	47	22	21	254	-	554	291	925
8	43	Male	2022.01	Pfizer	Pfizer	Pfizer	-	Pfizer	Pfizer	29	20	21	265	-	614	359	977
9	53	Female	2022.10	Pfizer	Pfizer	Pfizer	-	Pfizer	Pfizer	21	25	21	279	-	760	638	997
10	40	Female	2022.04	Pfizer	Pfizer	Pfizer	-	Moderna	Moderna	69	21	24	276	-	638	467	1003
11	37	Female	2022.05	Pfizer	Pfizer	Pfizer	-	Moderna	Moderna	8	20	21	280	-	643	508	1009
12	43	Female	2022.01	Pfizer	Pfizer	Pfizer	-	Moderna	Pfizer	3	32	21	234	-	566	309	913
13	36	Male	2022.08	Pfizer	Pfizer	Pfizer	-	Moderna	Moderna	2	30	21	316	-	655	583	980
14	59	Female	2022.04	Pfizer	Pfizer	Pfizer	Pfizer	Pfizer	Moderna	6	29	21	277	543	610	448	986
15	35	Female	2022.06	Pfizer	Pfizer	Pfizer	Pfizer	Pfizer	Pfizer	42	29	22	277	-	651	527	1008
subgroup 2: XBB infx + XBB.1.5 MV (n=10)																	
1	54	Female	2023.02	Pfizer	Pfizer	Pfizer	Moderna	Pfizer	Moderna	21	22	21	262	492	632	774	1003
2	62	Female	2023.04	Pfizer	Pfizer	Pfizer	Pfizer	Pfizer	Moderna	40	29	21	294	516	630	845	1000
3	61	Female	2023.04	Pfizer	Pfizer	Pfizer	Pfizer	Moderna	Moderna	53	26	21	275	519	625	843	1004
4	30	Female	2023.08	Pfizer	Pfizer	Pfizer	-	Moderna	Pfizer	21	22	21	218	-	580	922	880
5	58	Female	2023.02	Pfizer	Pfizer	Pfizer	-	Pfizer	Pfizer	10	22	21	284	-	682	1000	776
6	61	Male	2023.05	Pfizer	Pfizer	Moderna	-	Moderna	Moderna	7	30	21	231	-	541	917	800
7	42	Female	2023.03	Pfizer	Pfizer	Pfizer	Pfizer	Moderna	Pfizer	13	29	21	266	477	608	800	996
8	62	Female	2023.06	Pfizer	Pfizer	Pfizer	Pfizer	Pfizer	Pfizer	18	21	21	277	439	643	895	1002
9	46	Male	2023.05	J&J-Janssen	J&J-Janssen	Moderna	-	Pfizer	Pfizer	55	24	214	386	-	543	776	921
10	35	Female	2023.06	Moderna	Moderna	Moderna	Moderna	Moderna	Moderna	30	24	28	342	437	616	892	1014

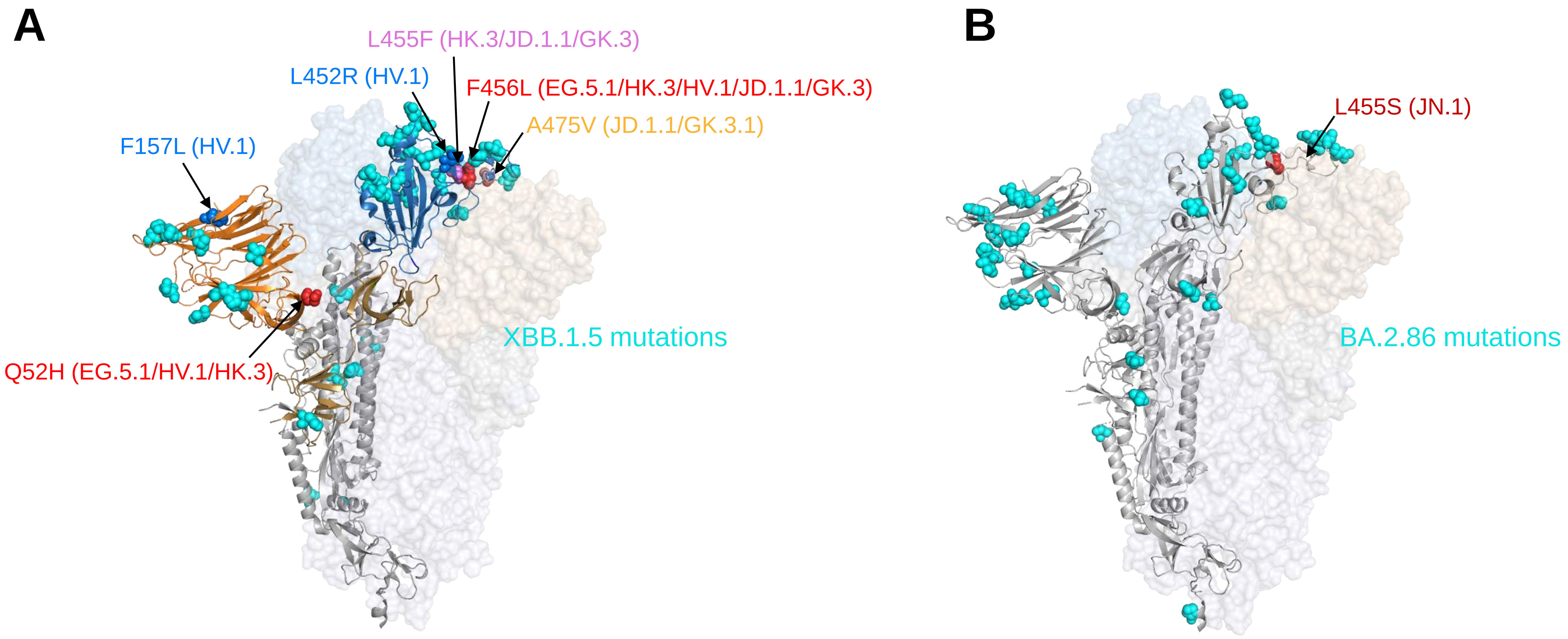

Figure S1. Spike mutations found in emerging SARS-CoV-2 Omicron subvariants, related to Figure 1.
A. Mutations found in EG.5.1, HV.1, HK.3, JD.1.1, and GK.3 on top of the XBB.1.5 spike.
B. Location of the L455S mutation in JN.1 on top of the BA.2.86 spike.
Mutations present in XBB.1.5 and BA.2.86 are highlighted in cyan. The spike protein structure is obtained under PDB ID: 6ZGE.

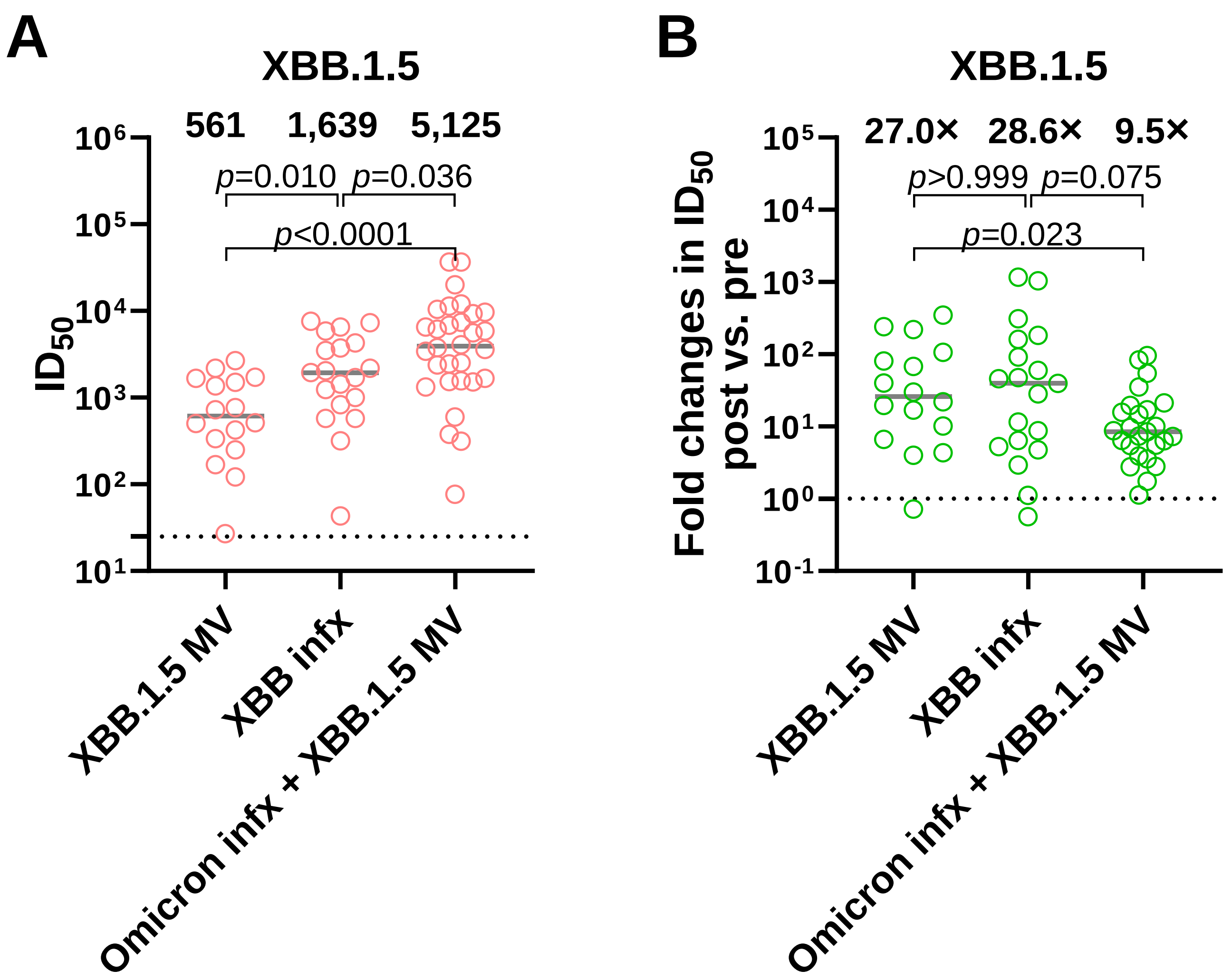

Figure S2. Neutralizing antibody titers against XBB.1.5 induced by XBB.1.5 monovalent vaccine, XBB infection or Omicron infection + XBB.1.5 monovalent vaccine. Related to Figure 2.

Serum neutralizing ID_{50} titers against XBB.1.5 of the indicated cohorts post vaccination or infection.

Fold increases in neutralizing ID_{50} titers of the indicated three cohorts against XBB.1.5 between pre and post vaccination or infection.

Geometric mean ID_{50} titers (**A**) and geometric mean fold changes (**B**) are shown above *p* values. Comparisons were made by Mann-Whitney unpaired *t* tests. The dotted lines represent the assay limit of detection of 25 (**A**) and fold change of 1 (**B**).

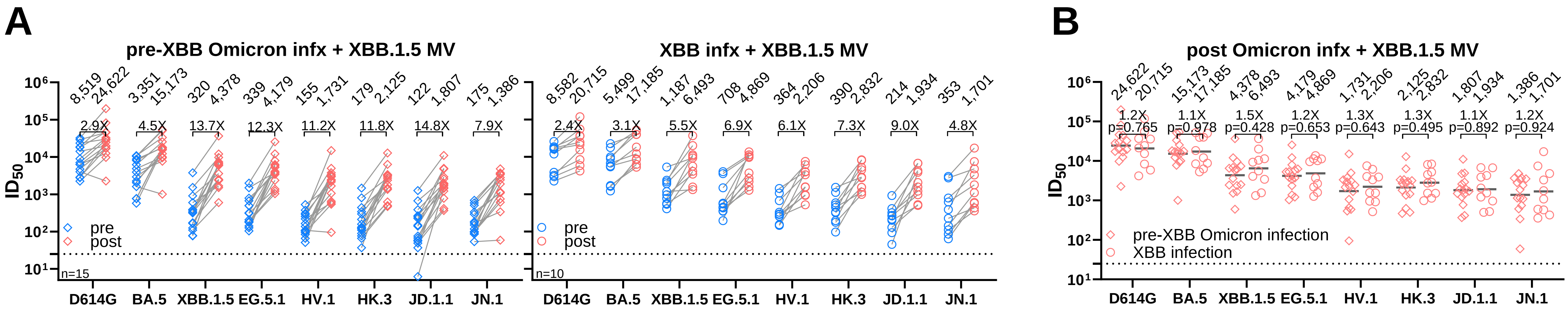

Figure S3. Neutralizing antibody titers of two subgroups of the "Omicron infx + XBB.1.5 MV" cohort, related to Figure 2.

A. Comparison in neutralizing antibody titers before and after an XBB.1.5 mRNA vaccine booster following a pre-XBB Omicron infection or an XBB infection. Geometric mean ID_{50} titers are shown along with the fold change between pre and post XBB.1.5 vaccination against each indicated virus.

B. Comparison in neutralizing antibody titers after an XBB.1.5 mRNA vaccine booster following a pre-XBB Omicron infection or an XBB infection. Statistical analyses were made by Mann-Whitney unpaired *t* tests. Geometric mean ID_{50} titers are shown along with the fold changes between the two subgroups.

Participants from the "Omicron infx + XBB.1.5 MV" cohort were stratified into two groups based on the infection strain. The dotted line represents the assay limit of detection of 25. "n" denotes the sample size.

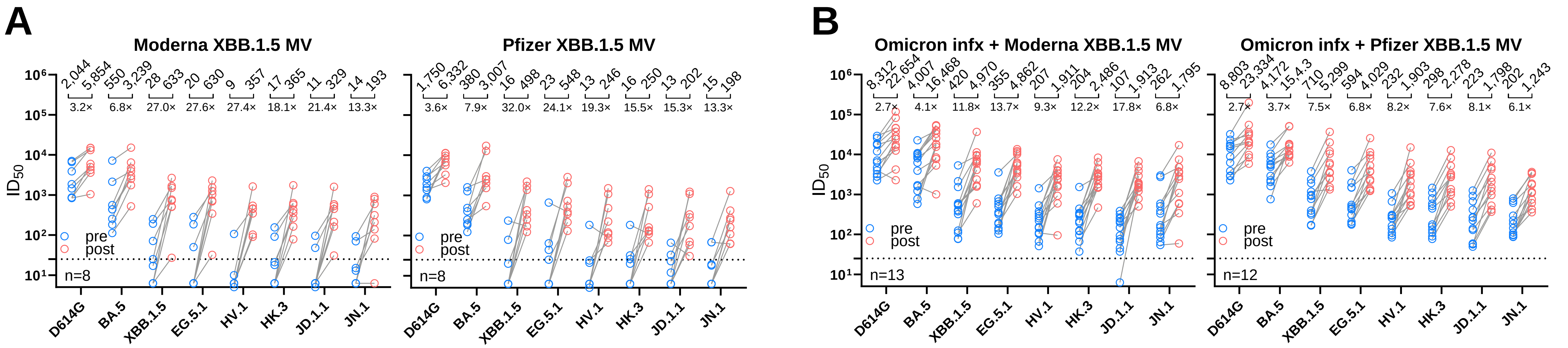

Figure S4. Neutralizing antibody titers before and after a Moderna or Pfizer XBB.1.5 mRNA vaccine booster, related to Figure 2. Participants from the "XBB.1.5 MV" cohort (**A**) and "Omicron infx + XBB.1.5 MV" (**B**) were stratified into two groups based on the vaccine manufacturer. Geometric mean ID_{50} titers are shown along with the fold change between pre and post XBB.1.5 vaccination against each indicated virus. The dotted line represents the assay limit of detection of 25. "n" denotes the sample size.
