# Supplementary material for: XBB.1.5 monovalent mRNA vaccine booster elicits robust neutralizing antibodies against XBB subvariants and JN.1
Source: Cell Host Microbe. 2024 Mar 13;32(3):315–321.e3. doi: 10.1016/j.chom.2024.01.014 (PMC10948033; doi:10.1016/j.chom.2024.01.014)
Supplement: Document S2. Article plus supplemental information [file mmc2.pdf]

Short article

Cell Host & Microbe

# XBB.1.5 monovalent mRNA vaccine booster elicits robust neutralizing antibodies against XBB subvariants and JN.1

## Graphical abstract

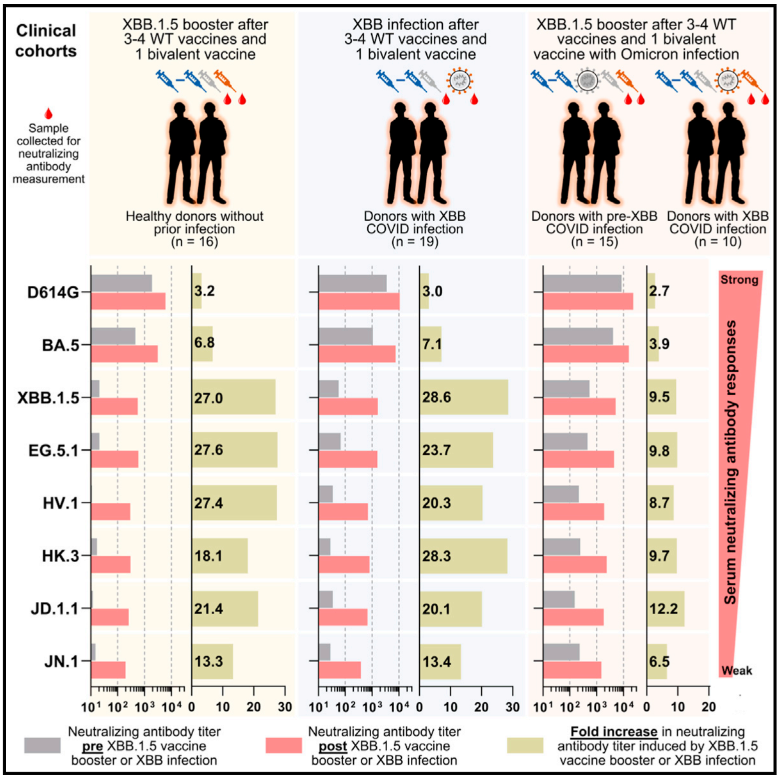


## Highlights

- An XBB.1.5 booster markedly enhanced neutralization of SARS-CoV-2, including JN.1
- An XBB.1.5 booster induced similar antibody titers to XBB infection
- Omicron infection followed by XBB.1.5 booster yielded the highest antibody titers
- Our findings supported recommendations for broad use of the updated XBB.1.5 booster


## Authors

Qian Wang, Yicheng Guo, Anthony Bowen, ..., Aubree Gordon, Lihong Liu, David D. Ho

## Correspondence

gordonal@umich.edu (A.G.), ll3411@cumc.columbia.edu (L.L.), dh2994@cumc.columbia.edu (D.D.H.)



## In brief

Wang et al. observed that administration of the updated COVID-19 vaccine booster targeting XBB.1.5 significantly increased neutralizing antibody responses against the most prevalent and emerging SARS-CoV-2 variants, including JN.1. This result was observed in both uninfected individuals and those with prior Omicron infection, supporting recommendations for its widespread use.


Wang et al., 2024, Cell Host & Microbe *32*, 315–321
March 13, 2024 © 2024 The Authors. Published by Elsevier Inc.
https://doi.org/10.1016/j.chom.2024.01.014

Cell Host & Microbe

CellPress OPEN ACCESS

Short article

# XBB.1.5 monovalent mRNA vaccine booster elicits robust neutralizing antibodies against XBB subvariants and JN.1

Qian Wang,[1,7] Yicheng Guo,[1,7] Anthony Bowen,[1,2,7] Ian A. Mellis,[1,3,7] Riccardo Valdez,[4] Carmen Gherasim,[4] Aubree Gordon,[5,*] Lihong Liu,[1,2,*] and David D. Ho[1,2,6,8,*]

[1]Aaron Diamond AIDS Research Center, Columbia University Vagelos College of Physicians and Surgeons, New York, NY 10032, USA

[2]Division of Infectious Diseases, Department of Medicine, Columbia University Vagelos College of Physicians and Surgeons, New York, NY 10032, USA

[3]Department of Pathology and Cell Biology, Columbia University Vagelos College of Physicians and Surgeons, New York, NY 10032, USA

[4]Department of Pathology, University of Michigan, Ann Arbor, MI 48109, USA

[5]Department of Epidemiology, University of Michigan, Ann Arbor, MI 48109, USA

[6]Department of Microbiology and Immunology, Columbia University Vagelos College of Physicians and Surgeons, New York, NY 10032, USA

[7]These authors contributed equally

[8]Lead contact

*Correspondence: gordonal@umich.edu (A.G.), ll3411@cumc.columbia.edu (L.L.), dh2994@cumc.columbia.edu (D.D.H.)

https://doi.org/10.1016/j.chom.2024.01.014

## SUMMARY

**COVID-19 vaccines have recently been updated to specifically encode or contain the spike protein of the SARS-CoV-2 XBB.1.5 subvariant, but their immunogenicity in humans has yet to be fully evaluated and reported, particularly against emergent viruses that are rapidly expanding. We now report that administration of an updated monovalent mRNA vaccine booster (XBB.1.5 MV) to previously uninfected individuals boosted serum virus-neutralizing antibodies significantly against not only XBB.1.5 (27.0-fold increase) and EG.5.1 (27.6-fold increase) but also key emerging viruses such as HV.1, HK.3, JD.1.1, and JN.1 (13.3- to 27.4-fold increase). Individuals previously infected by an Omicron subvariant had the highest overall serum neutralizing titers ($ID_{50}$ 1,504–22,978) against all viral variants tested. While immunological imprinting was still evident with the updated vaccines, it was not nearly as severe as observed with the previously authorized bivalent BA.5 vaccine. Our findings strongly support the official recommendation to widely apply the updated COVID-19 vaccines.**

## INTRODUCTION

Although the World Health Organization (WHO) has announced the conclusion of the emergency phase of the COVID-19 pandemic,[1] SARS-CoV-2 continues to spread and evolve.[2,3] Emerging viral variants increasingly evade host immunity acquired through vaccination, natural infection, or both, thereby posing a persistent threat to public health.[4] In particular, the emergence of Omicron XBB subvariants has dramatically reduced the efficacy of both SARS-CoV-2 wild-type monovalent and bivalent (wild type + Omicron BA.5) mRNA vaccines,[5] prompting the United States Food and Drug Administration (FDA) to authorize monovalent XBB.1.5-spike-based vaccines for individuals who are older than 6 months, starting in the fall of 2023.[6] Preliminary studies indicate that the updated monovalent vaccines substantially boosted serum virus-neutralizing antibody titers against previously dominant Omicron subvariants, such as XBB.1.5 and EG.5.1,[7–11] but their impact on viral variants that have subsequently emerged remains to be determined.

A number of SARS-CoV-2 Omicron subvariants have emerged recently, with several gaining traction in different parts of the globe (Figure 1A).[12] Until recently, over 90% of the new infections in Asia were attributed to JN.1, which has overtaken the previous HK.3 infection wave. Meanwhile, HV.1 constituted upward of 32% of the new cases in North America, but it is also being replaced by JN.1, now accounting for over 80%. In Europe, subvariants JD.1.1, BA.2.86, and JN.1 were all previously expanding, each accounting for 2.3%, 9.7%, and 75.0%, respectively, at the end of 2023, but there, too, JN.1 is becoming dominant, accounting for more than 85% of infections by the end of January 2024. HV.1, HK.3, and JD.1.1 have evolved from the XBB lineage, while JN.1 is a slight variant of BA.2.86,[2] which emerged independently from Omicron BA.2 (Figure 1B). Genetically, these subvariants have accumulated additional mutations in their spike proteins. By the end of 2023, it was clear that JN.1 has become globally dominant (Figure 1A).

Compared with EG.5.1, HK.3 possesses a unique mutation, L455F, while HV.1 carries two more mutations, F157L and


This is an open access article under the CC BY license (http://creativecommons.org/licenses/by/4.0/).

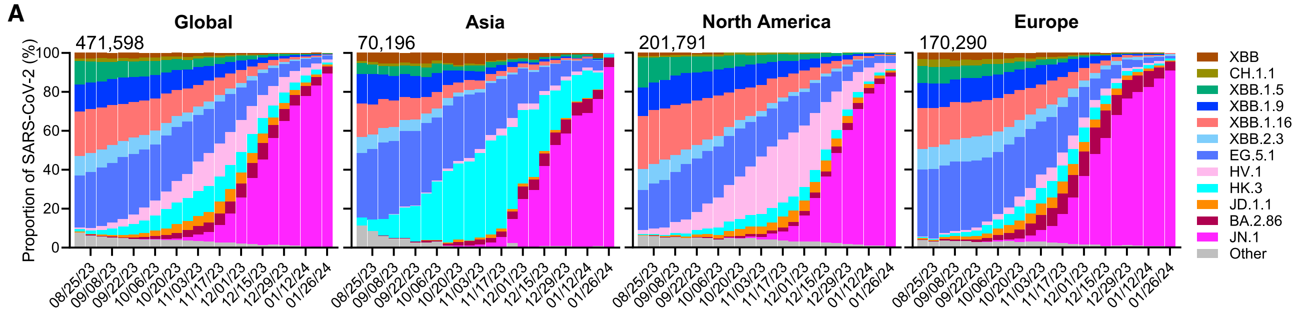


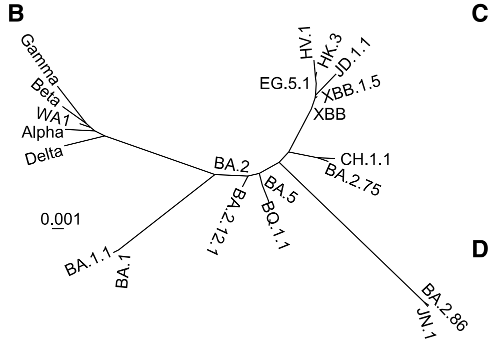


C

| D614G | T19 | L24 | P25 | P26 | A27 | Q52 | H69V70 | V83 | G142 | Y144 | H146 | F157 | Q183 | V213 | G252 | G339 | R346 | L368 | S371 | S373 | S375 | T376 | D405 | R408 | K417 | N440 | L452 | V445 | G446 | L452 | L455 | F456 | N460 | A475 | S477 | T478 | E484 | F486 | F490 | Q493 | Q498 | N501 | Y505 | H655 | N679 | P681 | N764 | D796 | Q954 | N969 |
|---|---|---|---|---|---|---|---|---|---|---|---|---|---|---|---|---|---|---|---|---|---|---|---|---|---|---|---|---|---|---|---|---|---|---|---|---|---|---|---|---|---|---|---|---|---|---|---|---|---|---|
| BA.2 | I | Δ | Δ | Δ | S | - | - | - | D | - | - | - | - | G | - | D | - | - | F | P | F | A | N | S | N | K | - | - | - | - | - | - | - | - | N | K | A | - | - | R | R | Y | H | Y | K | H | K | Y | H | K |
| BA.5 | I | Δ | Δ | Δ | S | - | Δ | - | D | - | - | - | - | G | - | D | - | - | F | P | F | A | N | S | N | K | R | - | - | - | - | - | - | - | N | K | A | V | - | Q | R | Y | H | Y | K | H | K | Y | H | K |
| XBB.1.5 | I | Δ | Δ | Δ | S | - | - | A | D | Δ | Q | - | E | E | V | H | T | I | F | P | F | A | N | S | N | K | - | P | S | - | - | - | K | - | N | K | A | P | S | Q | R | Y | H | Y | K | H | K | Y | H | K |
| EG.5.1 | I | Δ | Δ | Δ | S | H | - | A | D | Δ | Q | - | E | E | V | H | T | I | F | P | F | A | N | S | N | K | - | P | S | - | - | L | K | - | N | K | A | P | S | Q | R | Y | H | Y | K | H | K | Y | H | K |
| HK.3 | I | Δ | Δ | Δ | S | H | - | A | D | Δ | Q | - | E | E | V | H | T | I | F | P | F | A | N | S | N | K | - | P | S | - | F | L | K | - | N | K | A | P | S | Q | R | Y | H | Y | K | H | K | Y | H | K |
| HV.1 | I | Δ | Δ | Δ | S | H | - | A | D | Δ | Q | L | E | E | V | H | T | I | F | P | F | A | N | S | N | K | - | P | S | R | - | L | K | - | N | K | A | P | S | Q | R | Y | H | Y | K | H | K | Y | H | K |
| JD.1.1 | I | Δ | Δ | Δ | S | - | - | A | D | Δ | Q | - | E | E | V | H | T | I | F | P | F | A | N | S | N | K | - | P | S | - | F | L | K | V | N | K | A | P | S | Q | R | Y | H | Y | K | H | K | Y | H | K |

D

| BA.2 | V16ins | R21 | S50 | H69V70 | V127 | Y144 | F157 | R158 | N211 | L212 | L216 | H245 | A264 | I332 | D339 | K356 | R403 | V445 | G446 | N450 | L452 | L455 | N460 | N481 | V483 | A484 | F486 | R493 | E554 | A570 | P621 | H681 | S939 | P1143 |
|---|---|---|---|---|---|---|---|---|---|---|---|---|---|---|---|---|---|---|---|---|---|---|---|---|---|---|---|---|---|---|---|---|---|---|
| BA.2.86 | MP LF | T | L | Δ | F | Δ | S | G | Δ | I | F | N | D | V | H | T | K | H | S | D | W | - | K | K | Δ | K | P | Q | K | V | S | R | F | L |
| JN.1 | MP LF | T | L | Δ | F | Δ | S | G | Δ | I | F | N | D | V | H | T | K | H | S | D | W | S | K | K | Δ | K | P | Q | K | V | S | R | F | L |

**Figure 1. Emergence of novel SARS-CoV-2 variants**

(A) Frequencies of SAR-CoV-2 Omicron subvariants in the denoted time period. Data were obtained from the Global Initiative on Sharing All Influenza Data (GISAID).[12] The values in the upper left corner of each box denote the cumulative number of SAR-CoV-2 sequences deposited.

(B) Phylogenetic tree based on spike proteins of SARS-CoV-2 variants.

(C) Spike protein mutations in BA.2, BA.5, XBB.1.5, EG.5.1, HK.3, HV.1, and JD.1.1 relative to D614G.

(D) Spike protein mutations in BA.2.86 and JN.1 relative to BA.2.

See also Figure S1.

L452R (Figure 1C). JD.1.1 has three spike substitutions on top of those found in XBB.1.5, including the so-called "flip mutations" L455F and F456L as well as A475V. Moreover, JN.1 has an additional L455S mutation on the spike protein of BA.2.86 (Figure 1D). Interestingly, the aforementioned mutations reside predominantly in the class-1 epitope cluster[13] on the receptor-binding domain (RBD) of spike (Figure S1). In this study, we examined the outcome of an XBB.1.5 monovalent mRNA vaccine boost on serum neutralizing antibodies against these emerging and expanding SARS-CoV-2 Omicron subvariants.

## RESULTS

### Serum neutralization of emerging viral subvariants after an XBB.1.5 mRNA booster

To investigate the neutralizing antibody responses induced by XBB.1.5 mRNA monovalent vaccines against currently circulating and newly emerged subvariants, serum samples from 60 individuals across three different cohorts were collected. To accurately represent real-world conditions, all participants had previously received three to four doses of wild-type monovalent mRNA vaccines followed by one dose of a BA.5 bivalent mRNA vaccine. The three cohorts were (1) individuals with no recorded SARS-CoV-2 infections who received an XBB.1.5 monovalent vaccine booster ("XBB.1.5 MV"), (2) individuals with a recent XBB infection who did not receive an XBB.1.5 vaccine booster ("XBB infx"), and (3) individuals with a prior Omicron infection and had also received an XBB.1.5 monovalent vaccine booster ("Omicron infx + XBB.1.5 MV"). The final cohort was further divided into two subgroups: subgroup 1 with a documented infection before 2023 (pre-XBB Omicron infection) and subgroup 2 with a documented infection after February 2023 (XBB infection). Detailed demographics of study participants and their vaccination and infection histories are summarized in Tables S1 and S2. Figure 2A depicts the timeline of vaccine administration, SARS-CoV-2 infection, and serum collection for each cohort, and the time intervals between serum samples pre- and post-XBB.1.5 infection or monovalent vaccine boost are similar.

VSV-pseudotyped viruses were constructed for the emerging subvariants HV.1, HK.3, JD.1.1, and JN.1 as well as D614G, BA.5, XBB.1.5, EG.5.1 (Figures 1C and 1D). These pseudoviruses were then subjected to neutralization assays by pre- and post-XBB-exposure serum samples from the cohorts. In the XBB.1.5 MV cohort, the post-vaccination sera showed a 3.2-fold increase in neutralizing 50% inhibitory dilution ($ID_{50}$) titers against D614G and a 6.8-fold increase against BA.5, compared with pre-vaccination sera (Figure 2B). A larger increase in $ID_{50}$ titers was observed between pre- and post-vaccination sera against XBB.1.5, EG.5.1, HV.1, HK.3, JD.1.1, and JN.1, ranging from 13.3 to 27.6 fold. The magnitude of

CellPress
OPEN ACCESS

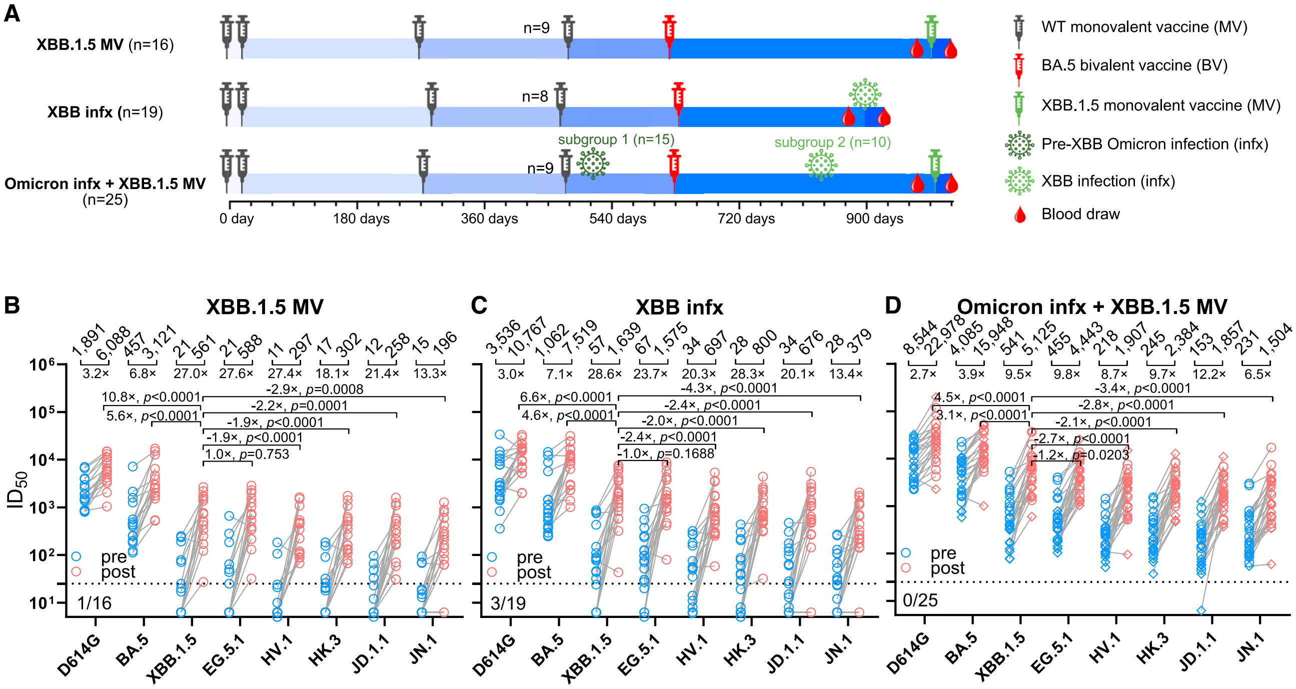

**Figure 2. Neutralizing antibody titers before and after an XBB.1.5 mRNA booster, XBB infection, or both**

(A) Timeline representation of vaccine administration, SARS-CoV-2 infection, and serum collection intervals for each clinical cohort. Indicated time points represent the median in days for each cohort, with day 0 defined as the day of the initial SARS-CoV-2 vaccination. Numbers of participants for each group receiving a fourth wild-type (WT) monovalent vaccine (MV) is indicated. Other vaccine doses were received by all participants in each cohort. 15 participants from the Omicron infx + XBB.1.5 MV cohort had a pre-XBB Omicron infection (subgroup 1), while the other 10 had XBB infection (subgroup 2). N, sample size.(B–D) Serum virus-neutralizing titers ($ID_{50}$) of the cohorts against the indicated SARS-CoV-2 pseudoviruses. Geometric mean $ID_{50}$ titers (GMT) are shown along with the fold change between pre and post (MV or infx) serum samples. Horizontal bars show the fold change in GMT following XBB MV or infection between XBB.1.5 and all other viruses tested. The dotted line represents the assay limit of detection (LOD) of 25. Numbers under the dotted lines are non-responders to XBB MV or infection (<3-fold increase in $ID_{50}$ titers between pre- and post-XBB sera across all the viruses tested). In the Omicron infx + XBB.1.5 MV cohort, subgroups 1 and 2 are shown in rhombuses and circles, respectively. Statistical analyses were performed by Wilcoxon matched-pairs signed-rank tests.
See also Tables S1 and S2 and Figures S2, S3, and S4.

these boosts was similar to those found for the XBB infx cohort (Figure 2C), which exhibited a 3.0-fold increase against D614G, a 7.1-fold increase against BA.5, and from 13.4- to 28.6-fold increases against XBB.1.5 and subsequent Omicron subvariants. Not surprisingly, sera from the Omicron infx + XBB.1.5 MV cohort displayed highest neutralization titers overall but smaller increases (Figures 2D and S2), largely attributable to higher titers in pre-vaccination samples due to a prior Omicron infection. Notably, the increase in neutralization activity following the XBB.1.5 monovalent vaccine booster was again much more pronounced against XBB.1.5 and newer Omicron subvariants (6.5- to12.2-fold increase) than against D614G and BA.5 (2.7 and 3.9 fold, respectively). Interestingly, no significant differences in neutralizing titers after vaccination were observed between subgroup 1 (pre-XBB Omicron group) and subgroup 2 (XBB Omicron group) (Figures 2D and S3). The potential reasons are that the antigenic distances to wild-type SARS-CoV-2 are relatively large for both the pre-XBB Omicron and XBB; in addition, pre-XBB Omicron and XBB are subvariants that are genetically close. This genetic closeness is particularly notable when compared with the vaccine components present in the monovalent and BA.5 bivalent mRNA vaccines. Furthermore, the administration of a booster with the XBB.1.5 monovalent vaccine, which has a large antigenic distance from the wild-type SARS-CoV-2 spike protein, may further minimize the differences in antibody responses observed between the two groups (Figure S3B).

After XBB.1.5 vaccination or infection across all three cohorts, the serum neutralization $ID_{50}$ titers against D614G were the highest, ranging from 6,088 to 22,978, followed by those against BA.5, ranging from 3,121 to 15,948 (Figures 2B–2D). Compared with BA.5, XBB.1.5 was significantly, i.e., 3.1- to 5.6-fold more resistant to neutralization by these sera, whereas it was minimally, i.e., 1.0- to 1.2-fold more sensitive than EG.5.1. Serum neutralization titers against newly emerged subvariants HV.1, HK.3, and JD.1.1 were quite similar but significantly, i.e, 1.9- to 2.8-fold lower than those against XBB.1.5. Overall, serum titers against JN.1 were the lowest—2.9- to 4.3-fold lower than the titers against XBB.1.5, which is expected given the exposure histories of these cohorts. Importantly, the absolute neutralization titers were robust against all viral variants tested for serum samples after XBB.1.5 vaccination or infection (Figures 2B–2D and S2), and the potency and breadth of the antibody boosts were similar for the two XBB.1.5 monovalent mRNA vaccines from different manufacturers, Moderna and Pfizer (Figures S4A and S4B).

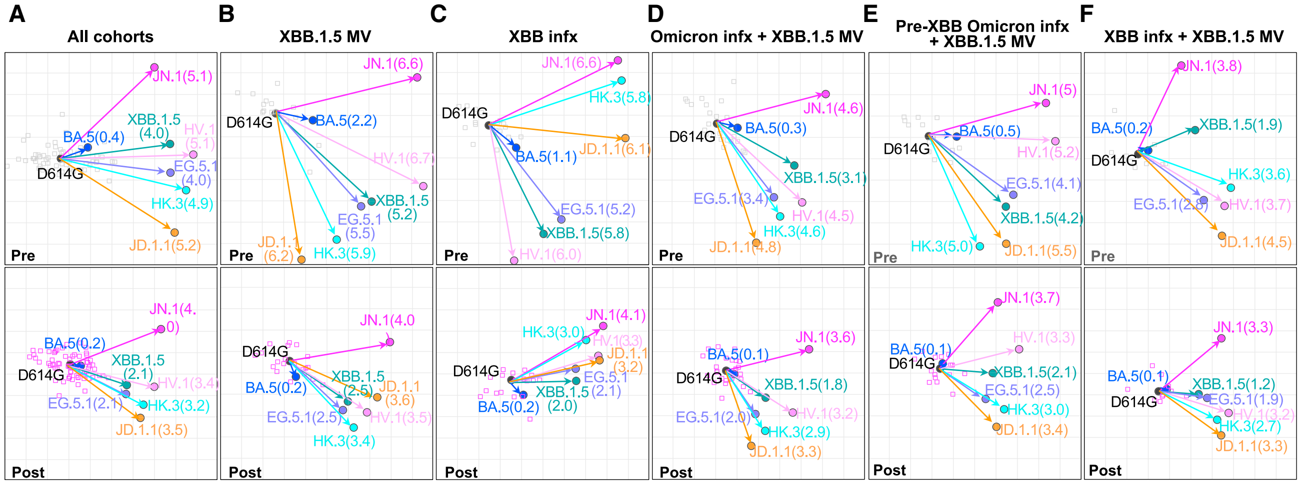


**Figure 3. Antigenic cartography of serum virus-neutralizing data**

Antigenic maps for all cohorts (A), the XBB.1.5 monovalent vaccine (XBB.1.5 MV) cohort (B), the XBB infection (XBB infx) cohort (C), the full infection + XBB.1.5 monovalent vaccine (Omicron infx + XBB.1.5 MV) cohort (D), and the pre-XBB and XBB subgroups of the Omicron infx + XBB.1.5 MV cohort (E and F). The top row shows antigenic maps generated with pre-XBB sera, and the bottom row shows maps generated with post-XBB sera. The length of each square in the antigenic maps corresponds to one antigenic unit and represents an approximately 2-fold change in $ID_{50}$ titer. Virus positions are shown in closed circles, while serum positions are shown by gray squares (pre-XBB sera) or pink squares (post-XBB sera). Antigenic distance from D614G is shown for each virus in parenthesis.

## Antigenic cartography

The serum neutralization data from all three cohorts combined, as well as individually, were used to construct antigenic maps (Figures 3A–3F), which graphically emphasize several key points. First, the discernible shortening of antigenic distances between D614G and other SARS-CoV-2 variants after XBB.1.5 monovalent vaccine administration (Figures 3B and 3D–3F) was indicative of the significant boost in antibody potency and breadth. Second, the shortening of these antigenic distances after XBB.1.5 infection was also similar (Figure 3C) to that after XBB.1.5 vaccine booster administration (Figure 3B), suggesting that infection and vaccination resulted in comparable enhancement of antibody responses. Third, the emergent subvariants HV.1, HK.3, and JD.1.1 clustered together but were more distant than XBB.1.5 and EG.5.1 (Figure 3), demonstrating not only their antigenic similarity but also their greater antibody resistance compared with their predecessors. Finally, JN.1 was antigenically distinct and more distant.

## Comparison of XBB.1.5 monovalent mRNA booster versus BA.5 bivalent mRNA booster

Following the XBB.1.5 monovalent vaccine booster administration, the highest neutralizing titers were observed against D614G and BA.5, not against XBB.1.5 (Figures 2B and 2D). This finding showed that there was considerable "back boosting" of antibodies directed to prior SARS-CoV-2 variants, which is likely the consequence of immunological imprinting[14] from prior vaccinations with the wild-type monovalent vaccine and the BA.5 bivalent vaccine. Nevertheless, an XBB.1.5 monovalent vaccine booster did markedly elevate serum neutralization titers against all Omicron subvariants tested (Figures 2B and 2D), in contrast to prior results obtained after the BA.5 bivalent vaccine booster dose.[15–19] We thus compared the relative increases in virus-neutralizing titers following the administration of either the XBB.1.5 monovalent vaccine or the BA.5 bivalent vaccine. Serum neutralization data against D614G, BA.5, and XBB.1.5, generated using assays identical to those described herein, were extracted from our previous report[18] on a cohort of individuals who received four injections of a wild-type monovalent vaccine followed by two injections of a BA.5 bivalent vaccine and then compared with the data extracted from two cohorts in the present study (Figure 4A). In individuals who received a second BA.5 bivalent booster, increases in mean serum neutralization titers against BA.5 were similar to those against D614G (2.6-fold versus 2.0-fold increase) (Figure 4B). However, strikingly, both the XBB.1.5 monovalent vaccine booster cohort (Figure 4C) and XBB breakthrough infection cohort (Figure 4D) showed markedly higher increases in mean neutralizing antibody titers against XBB.1.5 (27.0 and 28.6 fold, respectively) than against D614G (3.2 and 3.0 fold, respectively). These contrasting findings indicate that immunological imprinting is less severe for the XBB.1.5 monovalent vaccines.

## DISCUSSION

Our findings showed that both XBB.1.5 monovalent mRNA vaccine booster and XBB.1.5 breakthrough infection markedly increased the magnitude of serum neutralizing antibodies against SARS-CoV-2 Omicron subvariants such as XBB.1.5 and EG.5.1 (Figure 2), in general agreement with the clinical data posted by Chalkias et al.,[7] Marking et al.,[20] Kosugi et al.,[11] and Stankov et al.[8] and the animal immunization results posted by Patel et al.[9] and Modjarrad et al.[10] The latter three studies also found that there are strong specific T cell responses directed to the spike protein of XBB subvariants.[8–10] Here, we extended our study to include the emerging Omicron subvariants that are now gaining traction and expanding rapidly, including HV.1, HK.3, JD.1.1, which are descendants of the

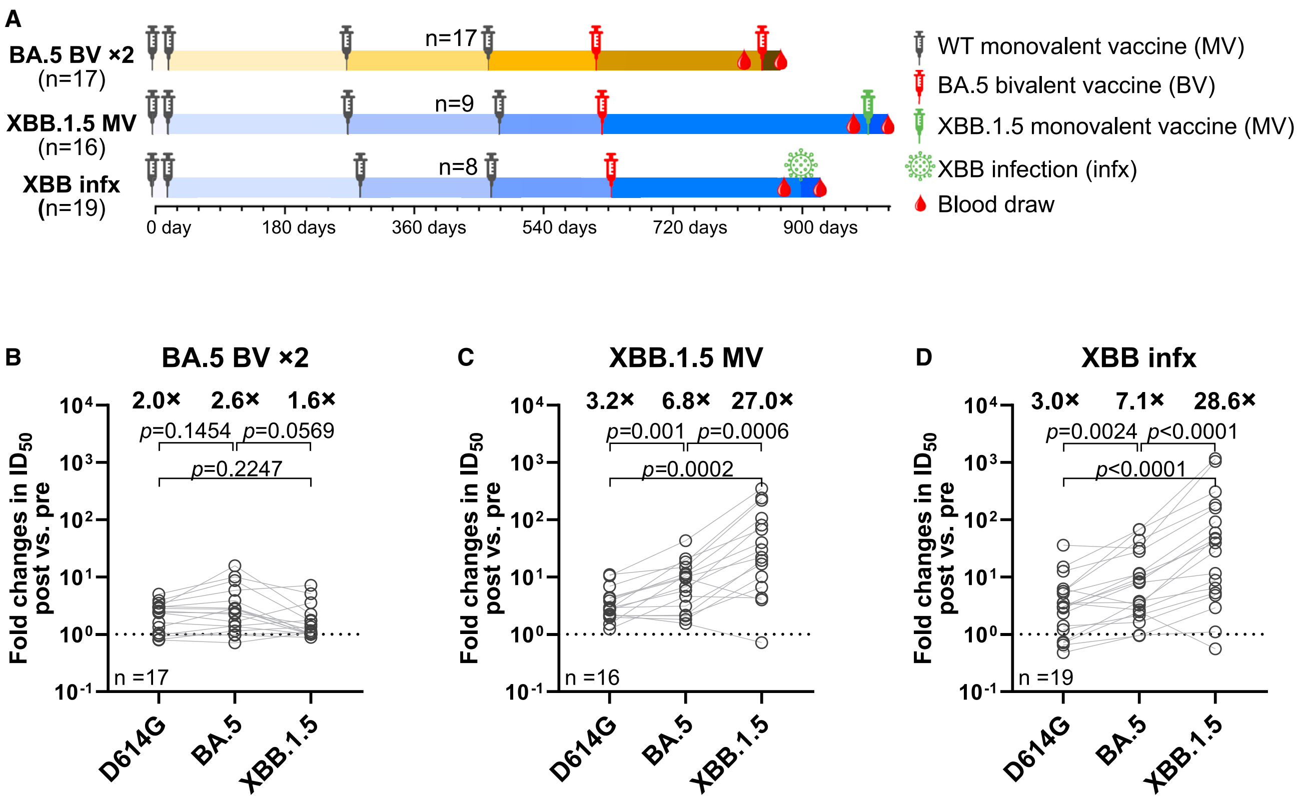


**Figure 4. XBB.1.5 monovalent mRNA vaccines induced stronger boosts than a second BA.5 bivalent mRNA vaccine**

(A) Timeline representation of vaccine administration, SARS-CoV-2 infection, and serum collection intervals for each cohort.[18] The cohort that received a second BA.5 bivalent vaccine (BA.5 BV × 2) was previously described. Indicated timepoints represent the median in days for each cohort, with day 0 defined as the day of the initial SARS-CoV-2 vaccination. Numbers of participants for each group receiving a fourth wild-type (WT) monovalent vaccine is indicated. n, sample size.
(B–D) Fold changes in $ID_{50}$ titers of the indicated cohorts against D614G, BA.5, and XBB.1.5 between pre- and post-vaccination or infection. Geometric mean fold changes in $ID_{50}$ titer are shown as black bars and denoted above the dots. Statistical analyses were performed by employing Wilcoxon matched-pairs signed-rank tests. Data for the BA.5 BV × 2 cohort were extracted from a previously published study.[18]

XBB lineage, as well as the currently dominant JN.1, which is closely related to BA.2.86 (Figure 1B). Serum neutralizing titers against these emergent viruses increased by ~13–27 fold after an XBB.1.5 monovalent vaccine booster administration in individuals without an infection history (Figure 2B) and by ~10 fold in individuals with a prior Omicron infection (Figure 2D) Interestingly, we also showed that those who were administered an XBB.1.5 monovalent vaccine booster dose elicited serum neutralization potency and breadth similar to those with an XBB.1.5 breakthrough infection (Figures 2B, 2C, 3B, and 3C).

Our results also showed that HV.1, HK.3, and JD.1.1 are 1.9- to 2.8-fold more resistant to serum neutralization than XBB.1.5 (Figures 2B–2D), a finding that suggests that these emergent subvariants are likely to have a growth advantage in the population over their immediate precursors. If so, we can expect these new sublineages to replace XBB.1.5 and EG.5.1. Likewise, JN.1 is even more antibody resistant, by 2.9–4.3 fold, to the serum samples tested here (Figure 2). Therefore, while our data support the recommendation of XBB.1.5 MV vaccine to the public, widespread application of the updated XBB.1.5 monovalent vaccines could confer an even larger growth advantage in the population to JN.1 as well as to the related BA.2.86. This could potentially lead to the emergence of subvariants that are more evasive to antibodies, thereby posing a potential threat to the newly authorized COVID-19 vaccines.

Our findings suggest that immunological imprinting is evident with the XBB.1.5 monovalent mRNA vaccines studied, in concordance with the findings by Tortorici et al.[21] However, as discussed above, it is not nearly as severe as those observed for the BA.5 bivalent vaccines (Figure 4). One potential explanation is that XBB.1.5 is genetically and antigenically more distant from the ancestral SARS-CoV-2 than BA.5, which might mitigate immunological imprinting to an extent. Perhaps a more likely explanation is the non-inclusion of the ancestral spike in the current XBB.1.5 monovalent vaccines. Previous studies on the bivalent WA1 + BA5 vaccines by our team[15–18] and others[19] suggested that the inclusion of the ancestral spike exacerbated the problem of imprinting and recommended its removal. Our findings herein indicate that WHO, FDA, and the vaccine manufacturers made the right choice by formulating the new COVID-19 vaccines based on XBB.1.5 spike alone, without including the ancestral spike.

## Limitations of the study

This study is limited to the evaluation of serum neutralizing antibodies, without addressing T cell responses[22–24] or mucosal

immunity,[25–27] both of which could provide added protection against SARS-CoV-2. Moreover, studies evaluating binding antibody responses and variant-specific B cell frequencies could provide a more comprehensive understanding of immune imprinting. Lastly, we have only examined acute antibody responses after XBB.1.5 monovalent vaccine booster or XBB.1.5 infection, but how such responses evolve over time will require follow-up studies. Despite these limitations, our results demonstrate that the administration of an XBB.1.5 monovalent mRNA vaccine booster can elicit robust neutralizing antibodies against current and emerging SARS-CoV-2 variants. This study includes cohorts composed of adult male and female participants (78.3% female). However, due to sample size and study design constraints, we do not report analysis of male- or female-specific results. Furthermore, our findings support FDA's recommendation to apply these updated COVID-19 vaccines more widely to confer greater protection to the public.

## STAR★METHODS

Detailed methods are provided in the online version of this paper and include the following:

- KEY RESOURCES TABLE
- RESOURCE AVAILABILITY
  - Lead contact
  - Materials availability
  - Data and code availability
- EXPERIMENTAL MODEL AND SUBJECT DETAILS
  - Clinical cohorts
  - Cell lines
- METHOD DETAILS
  - Pseudovirus neutralization assay
  - Phylogenetic analysis
  - Antigenic cartography
- QUANTIFICATION AND STATISTICAL ANALYSIS

## SUPPLEMENTAL INFORMATION

Supplemental information can be found online at https://doi.org/10.1016/j.chom.2024.01.014.

## ACKNOWLEDGMENTS

This study was supported by funding from the NIH SARS-CoV-2 Assessment of Viral Evolution (SAVE) Program (subcontract no. 0258-A709-4609 under federal contract no. 75N93021C00014) and the Gates Foundation (project INV019355) to D.D.H., as well as funding from NIH contract 75N9301 9C00051 to A.G. We express our gratitude to Zijin Chu, Theresa Kowalski-Dobson, Emily Stoneman, David Manthei, Anna Buswinka, Gabe Simjanovski, Joseph Wendzinski, Mayurika Patel, Kathleen Lindsey, Dawson Davis, Victoria Blanc, Savanna Sneeringer, and Pamela Bennett-Baker of the IASO study team for conducting the IASO study.

## AUTHOR CONTRIBUTIONS

The study was conceptualized by A.G., L.L., and D.D.H. Experiments were conducted and data were analyzed by Q.W., L.L., Y.G., I.A.M., and A.B. Project management was handled by Q.W. Serum samples were collected by R.V., C.G., A.G., and their colleagues. The results were analyzed and the manuscript was written by Q.W., Y.G., L.L., and D.D.H. All contributing authors have reviewed and endorsed the manuscript.

## DECLARATION OF INTERESTS

D.D.H. co-founded TaiMed Biologics and RenBio, serves as a consultant for WuXi Biologics and Brii Biosciences, and is a board director at Vicarious Surgical. A.G. served as a member of the scientific advisory board for Janssen Pharmaceuticals.

Received: December 5, 2023
Revised: January 4, 2024
Accepted: January 26, 2024
Published: February 19, 2024

## REFERENCES

1. WHO (2023). Statement on the fifteenth meeting of the IHR (2005) Emergency Committee on the COVID-19 pandemic. https://www.who.int/news/item/05-05-2023-statement-on-the-fifteenth-meeting-of-the-international-health-regulations-(2005)-emergency-committee-regarding-the-coronavirus-disease-(covid-19)-pandemic.
2. Wang, Q., Guo, Y., Liu, L., Schwanz, L.T., Li, Z., Nair, M.S., Ho, J., Zhang, R.M., Iketani, S., Yu, J., et al. (2023). Antigenicity and receptor affinity of SARS-CoV-2 BA.2.86 spike. Nature *624*, 639–644.
3. Wang, Q., Guo, Y., Zhang, R.M., Ho, J., Mohri, H., Valdez, R., Manthei, D.M., Gordon, A., Liu, L., and Ho, D.D. (2023). Antibody neutralisation of emerging SARS-CoV-2 subvariants: EG.5.1 and XBC.1.6. Lancet Infect. Dis. *23*, e397–e398.
4. Carabelli, A.M., Peacock, T.P., Thorne, L.G., Harvey, W.T., Hughes, J., COVID-19 Genomics UK Consortium, Peacock, S.J., Barclay, W.S., de Silva, T.I., Towers, G.J., et al. (2023). SARS-CoV-2 variant biology: immune escape, transmission and fitness. Nat. Rev. Microbiol. *21*, 162–177.
5. Wang, Q., Iketani, S., Li, Z., Liu, L., Guo, Y., Huang, Y., Bowen, A.D., Liu, M., Wang, M., Yu, J., et al. (2023). Alarming antibody evasion properties of rising SARS-CoV-2 BQ and XBB subvariants. Cell *186*, 279–286.
6. FDA (2023). Updated COVID-19 Vaccines for Use in the United States Beginning in Fall 2023. https://www.fda.gov/vaccines-blood-biologics/updated-covid-19-vaccines-use-united-states-beginning-fall-2023.
7. Chalkias, S., McGhee, N., Whatley, J.L., Essink, B., Brosz, A., Tomassini, J.E., Girard, B., Wu, K., Edwards, D.K., Nasir, A., et al. (2023). Safety and Immunogenicity of XBB.1.5-Containing mRNA Vaccines. Preprint at medRxiv. https://doi.org/10.1101/2023.08.22.23293434.
8. Stankov, M.V., Hoffmann, M., Gutierrez Jauregui, R., Cossmann, A., Morillas Ramos, G., Graalmann, T., Winter, E.J., Friedrichsen, M., Ravens, I., Ilievska, T., et al. (2024). Humoral and cellular immune responses following BNT162b2 XBB.1.5 vaccination. Lancet Infect. Dis. *24*, e1–e3.
9. Patel, N., Trost, J.F., Guebre-Xabier, M., Zhou, H., Norton, J., Jiang, D., Cai, Z., Zhu, M., Marchese, A.M., Greene, A.M., et al. (2023). XBB.1.5 spike protein COVID-19 vaccine induces broadly neutralizing and cellular immune responses against EG.5.1 and emerging XBB variants. Sci. Rep. *13*, 19176.
10. Modjarrad, K., Che, Y., Chen, W., Wu, H., Cadima, C.I., Muik, A., Maddur, M.S., Tompkins, K.R., Martinez, L.T., Cai, H., et al. (2023). Preclinical Characterization of the Omicron XBB.1.5-Adapted BNT162b2 COVID-19 Vaccine. Preprint at bioRxiv. https://doi.org/10.1101/2023.11.17.567633.
11. Kosugi, Y., Kaku, Y., Hinay, A.A., Jr., Guo, Z., Uriu, K., Kihara, M., Saito, F., Uwamino, Y., Kuramochi, J., Shirakawa, K., et al. (2024). Antiviral humoral immunity against SARS-CoV-2 omicron subvariants induced by XBB.1.5 monovalent vaccine in infection-naive and XBB-infected individuals. Lancet Infect. Dis. https://doi.org/10.1016/S1473-3099(23)00784-3.
12. Elbe, S., and Buckland-Merrett, G. (2017). Data, disease and diplomacy: GISAID's innovative contribution to global health. Glob. Chall. *1*, 33–46.
13. Barnes, C.O., Jette, C.A., Abernathy, M.E., Dam, K.A., Esswein, S.R., Gristick, H.B., Malyutin, A.G., Sharaf, N.G., Huey-Tubman, K.E., Lee, Y.E., et al. (2020). SARS-CoV-2 neutralizing antibody structures inform therapeutic strategies. Nature *588*, 682–687.

CellPress
OPEN ACCESS

14. Koutsakos, M., and Ellebedy, A.H. (2023). Immunological imprinting: understanding COVID-19. Immunity *56*, 909–913.

15. Wang, Q., Guo, Y., Tam, A.R., Valdez, R., Gordon, A., Liu, L., and Ho, D.D. (2023). Deep immunological imprinting due to the ancestral spike in the current bivalent COVID-19 vaccine. Cell Rep. Med. *4*, 101258.

16. Wang, Q., Bowen, A., Valdez, R., Gherasim, C., Gordon, A., Liu, L., and Ho, D.D. (2023). Antibody response to omicron BA.4-BA.5 bivalent booster. N. Engl. J. Med. *388*, 567–569.

17. Wang, Q., Bowen, A., Tam, A.R., Valdez, R., Stoneman, E., Mellis, I.A., Gordon, A., Liu, L., and Ho, D.D. (2023). SARS-CoV-2 neutralising antibodies after bivalent versus monovalent booster. Lancet Infect. Dis. *23*, 527–528.

18. Wang, Q., Bowen, A., Ho, J., Zhang, R.M., Valdez, R., Stoneman, E., Gordon, A., Liu, L., and Ho, D.D. (2023). SARS-CoV-2 neutralising antibodies after a second BA.5 bivalent booster. Lancet *402*, 1827–1828.

19. Collier, A.Y., Miller, J., Hachmann, N.P., McMahan, K., Liu, J., Bondzie, E.A., Gallup, L., Rowe, M., Schonberg, E., Thai, S., et al. (2023). Immunogenicity of BA.5 bivalent mRNA vaccine boosters. N. Engl. J. Med. *388*, 565–567.

20. Marking, U., Bladh, O., Aguilera, K., Yang, Y., Greilert Norin, N., Blom, K., Hober, S., Klingström, J., Havervall, S., Åberg, M., et al. (2024). Humoral immune responses to the monovalent XBB.1.5-adapted BNT162b2 mRNA booster in Sweden. Lancet Infect. Dis. *24*, e80–e81.

21. Tortorici, M.A., Addetia, A., Seo, A.J., Brown, J., Sprouse, K.R., Logue, J., Clark, E., Franko, N., Chu, H., and Veesler, D. (2023). Persistent immune imprinting after XBB.1.5 COVID vaccination in humans. Preprint at bioRxiv. https://doi.org/10.1101/2023.11.28.569129.

22. Sette, A., and Crotty, S. (2021). Adaptive immunity to SARS-CoV-2 and COVID-19. Cell *184*, 861–880.

23. Zhang, Z., Mateus, J., Coelho, C.H., Dan, J.M., Moderbacher, C.R., Gálvez, R.I., Cortes, F.H., Grifoni, A., Tarke, A., Chang, J., et al. (2022). Humoral and cellular immune memory to four COVID-19 vaccines. Cell *185*, 2434–2451.e17.

24. Vogel, A.B., Kanevsky, I., Che, Y., Swanson, K.A., Muik, A., Vormehr, M., Kranz, L.M., Walzer, K.C., Hein, S., Güler, A., et al. (2021). BNT162b vaccines protect rhesus macaques from SARS-CoV-2. Nature *592*, 283–289.

25. Tang, J., Zeng, C., Cox, T.M., Li, C., Son, Y.M., Cheon, I.S., Wu, Y., Behl, S., Taylor, J.J., Chakaraborty, R., et al. (2022). Respiratory mucosal immunity against SARS-CoV-2 after mRNA vaccination. Sci. Immunol. *7*, eadd4853.

26. Afkhami, S., D'Agostino, M.R., Zhang, A., Stacey, H.D., Marzok, A., Kang, A., Singh, R., Bavananthasivam, J., Ye, G., Luo, X., et al. (2022). Respiratory mucosal delivery of next-generation COVID-19 vaccine provides robust protection against both ancestral and variant strains of SARS-CoV-2. Cell *185*, 896–915.

27. Mao, T., Israelow, B., Peña-Hernández, M.A., Suberi, A., Zhou, L., Luyten, S., Reschke, M., Dong, H., Homer, R.J., Saltzman, W.M., et al. (2022). Unadjuvanted intranasal spike vaccine elicits protective mucosal immunity against sarbecoviruses. Science *378*, eabo2523.

28. Wang, Q., Guo, Y., Iketani, S., Nair, M.S., Li, Z., Mohri, H., Wang, M., Yu, J., Bowen, A.D., Chang, J.Y., et al. (2022). Antibody evasion by SARS-CoV-2 Omicron subvariants BA.2.12.1, BA.4 and BA.5. Nature *608*, 603–608.

29. Simon, V., Kota, V., Bloomquist, R.F., Hanley, H.B., Forgacs, D., Pahwa, S., Pallikkuth, S., Miller, L.G., Schaenman, J., Yeaman, M.R., et al. (2022). Paris and SPARTA: finding the Achilles' heel of SARS-CoV-2. mSphere *7*, e0017922.

30. Smith, D.J., Lapedes, A.S., de Jong, J.C., Bestebroer, T.M., Rimmelzwaan, G.F., Osterhaus, A.D., and Fouchier, R.A. (2004). Mapping the antigenic and genetic evolution of influenza virus. Science *305*, 371–376.

## STAR★METHODS

### KEY RESOURCES TABLE

| REAGENT or RESOURCE | SOURCE | IDENTIFIER |
|---|---|---|
| Bacterial and virus strains | | |
| VSV-G pseudotyped ΔG-luciferase | Kerafast | Cat# EH1020-PM |
| Biological samples | | |
| "XBB.1.5 MV" sera | This paper | N/A |
| "XBB infx" sera | This paper | N/A |
| "Omicron infx + XBB.1.5 MV" sera | This paper | N/A |
| "BA.5 BV ×2" sera | Wang et al.[18] | N/A |
| Chemicals, peptides, and recombinant proteins | | |
| Polyethylenimine (PEI) | Polysciences Inc. | Cat# 23966-100 |
| Critical commercial assays | | |
| Luciferase Assay System | Promega | Cat# E4550 |
| QuikChange Lightning Site-Directed Mutagenesis Kit | Agilent | Cat# 210518 |
| Experimental models: cell lines | | |
| HEK293T | ATCC | Cat# CRL-3216; RRID: CVCL_0063 |
| Vero-E6 | ATCC | Cat# CRL-1586; RRID: CVCL_0574 |
| Recombinant DNA | | |
| pCMV3-D614G | Wang et al.[28] | N/A |
| pCMV3-BA.5 | Wang et al.[28] | N/A |
| pCMV3-XBB.1.5 | Wang et al.[17] | N/A |
| pCMV3-EG.5.1 | Wang et al.[3] | N/A |
| pCMV3-HV.1 | This paper | N/A |
| pCMV3-HK.3 | This paper | N/A |
| pCMV3-JD.1.1 | This paper | N/A |
| pCMV3-JN.1 | This paper | N/A |
| Software and algorithms | | |
| GraphPad Prism 10 | GraphPad Software Inc | https://www.graphpad.com/scientific-software/prism/ |
| MUSCLE V3.8.31 | N/A | https://bioweb.pasteur.fr/packages/pack@muscle@3.8.31 |
| MEGA11 | N/A | https://www.megasoftware.net/home |

## RESOURCE AVAILABILITY

### Lead contact

Further information and requests for resources and reagents should be directed to and will be fulfilled by the lead contact, David D. Ho (dh2994@cumc.columbia.edu).

### Materials availability

All reagents generated in this study are available from the lead contact with a completed materials transfer agreement.

### Data and code availability

Data reported in this paper will be shared by the lead contact upon request.

- This paper does not report original code.
- Any additional information required to reanalyze the data reported in this paper is available from the lead contact upon request.

## EXPERIMENTAL MODEL AND SUBJECT DETAILS

### Clinical cohorts

Longitudinal sera were obtained as part of a continuing cohort study, Immunity-Associated with SARS-CoV-2 Study (IASO), which began in 2020 at the University of Michigan in Ann Arbor, Michigan.[29] Written informed consent was provided by all participants and sera were collected according to the protocol approved by the Institutional Review Board of the University of Michigan Medical School. Participants in the IASO study completed weekly symptom surveys and were tested for SARS-CoV-2 with any report of symptoms. All serum samples were examined by anti-nucleoprotein (NP) ELISA to confirm status of prior SARS-CoV-2 infection.

For this study, we included sera from 60 individuals in three distinct clinical cohorts: 1) individuals with no recorded SARS-CoV-2 infections who had received an XBB.1.5 monovalent vaccine booster (“XBB.1.5 MV”); 2) individuals with a recent XBB SARS-CoV-2 infection who had not received the XBB.1.5 booster (“XBB infx″); and 3) individuals with prior infection who also received the XBB.1.5 booster (“Omicron infx + XBB.1.5 MV”). The final cohort was divided into subgroup 1, with documented infection prior to 2023, and subgroup 2, with documented infection after February 2023. Individuals in all cohorts received either three or four doses of a wildtype monovalent vaccine as well as a single BA.5 bivalent booster.

Most participants were female (78.3%) with an average age of 49.7 years. Age, sex, infection, and vaccination history are reported for each patient (Table S2). However, ancestry, race, ethnicity, and socioeconomic status are not reported. Sera were collected an average of 26 days pre and post XBB.1.5 vaccination or XBB infection. Sera were examined by anti-nucleoprotein (NP) ELISA to determine status of prior SARS-CoV-2 infection. Demographic, vaccination, and serum collection details are summarized for each cohort and subgroup in Table S1, and details are shown for each participant in Table S2.

### Cell lines

293T (CRL-3216) and Vero-E6 (CRL-1586) cells were obtained from ATCC and cultured in the conditions following manufacturer’s instructions. The morphology of each cell line was visually confirmed before use. All cell lines tested negative for mycoplasma.

## METHOD DETAILS

### Pseudovirus neutralization assay

Plasmids encoding SARS-CoV-2 variant spikes, including D614G, BA.5, XBB.1.5, and EG.5.1, were generated in previous studies.[3,5,17,28] Plasmids expressing HV.1, HK.3, JD.1.1, and JN.1 spikes were generated by introducing mutations to the XBB.1.5,[17] EG.5.1,[3] or BA.2.86[2] spike (Figure 1C) using the QuikChange® mutagenesis kit.

To produce pseudotyped viruses of SARS-CoV-2 variants, 293T cells were transfected with the spike-encoding plasmids described above using 1 mg/mL PEI (Polyethylenimine). One day post-transfection, the 293T cells were then incubated with VSVG*ΔG-luciferase (Kerafast, Inc.) at a multiplicity of approximately 3 to 5 for 2 h followed by three washes with PBS. The cells were then cultured with fresh medium for an additional day. Cell supernatants containing viruses were collected, clarified by centrifugation, aliquoted, and stored at -80°C until use.

The viral titer of each variant was titrated and normalized for the neutralization assays. Serum samples were diluted in triplicate in 96-well plates, starting from a 12.5-fold dilution, and then incubated with an equal volume of virus for 1 h at 37°C before adding $2 \times 10^4$ cells/well of Vero-E6 cells. The cells were then cultured overnight, harvested, and lysed for measurement of luciferase activity using SoftMax Pro v.7.0.2 (Molecular Devices). Reductions in luciferase activity at given dilutions of sera were calculated, and $ID_{50}$ values of sera were obtained by fitting the virus-reduction data using a non-linear five-parameter dose-response curve in GraphPad Prism V.10.

### Phylogenetic analysis

Genome sequences of SARS-CoV-2 subvariants are retrieved from the GISAID database.[12] The spike protein sequences are then extracted from these genomes using an in-house Python script. Post-extraction, these sequences are aligned by MUSCLE software, version 3.8.31. Sequencing sites with low quality, identified by the presence of ’N’, underwent a manual curation to align the mutations with the consensus for each variant. A Maximum-Likelihood phylogenetic tree was constructed with MEGA11 software, utilizing the Tamura-Nei model, and its robustness was verified through 500 bootstrap replications.

### Antigenic cartography

The antigenic distances between serum samples and D614G, along with other SARS-CoV-2 variants, were calculated by integrating the $ID_{50}$ values of individual serum samples using a published antigenic cartography method.[30] Visualizations are created with the Racmacs package (version 1.1.4, https://acorg.github.io/Racmacs/) within R software version 4.0.3. The optimization is set to 2,000 steps, with the “minimum column basis” parameter set to “none”. The “mapDistances” function was used to calculate the antigenic distances, with the average distances from all serum samples to each variant representing the final outputs. For each group, D614G was positioned as the center point of the sera. The seeds for each antigenic map are manually adjusted to position D614G left horizontally in relation to other variants.

## QUANTIFICATION AND STATISTICAL ANALYSIS

Serum neutralization $ID_{50}$ values were calculated using a five-parameter dose-response curve in GraphPad Prism v.10. Evaluations of statistical significance were performed employing either two-tailed Wilcoxon matched-pairs signed-rank tests or Mann-Whitney unpaired t tests using GraphPad Prism v.10 software.

Cell Host & Microbe, Volume *32*

## Supplemental information

# XBB.1.5 monovalent mRNA vaccine booster elicits robust neutralizing antibodies against XBB subvariants and JN.1

**Qian Wang, Yicheng Guo, Anthony Bowen, Ian A. Mellis, Riccardo Valdez, Carmen Gherasim, Aubree Gordon, Lihong Liu, and David D. Ho**

**Table S1. Summarized participant information, related to Figure 2.** Demographic, vaccine, and sera collection information is summarized for each cohort. Listed values represent the mean and range (age and sera collection variables) or number and percentage (vaccine type and sex variables).

| Clinical information | | All participants | | XBB.1.5 MV | | XBB infx | | Prior Infx + XBB MV | | | |
|---|---|---|---|---|---|---|---|---|---|---|---|
| | | | | | | | | Pre-XBB infx + XBB.1.5 MV | | XBB infx + XBB.1.5 MV | |
| | | No. or Mean | % or (range) | No. or Mean | % or (range) | No. or Mean | % or (range) | No. or Mean | % or (range) | No. or Mean | % or (range) |
| | **Total case** | 60 | - | 16 | - | 19 | - | 15 | - | 10 | - |
| | **Female** | 47 | 78.3% | 11 | 68.8% | 16 | 84.2% | 12 | 80.0% | 8 | 80.0% |
| | **Male** | 13 | 21.7% | 5 | 31.3% | 3 | 15.8% | 3 | 20.0% | 2 | 20.0% |
| | **Age** | 49.7 | (30,77) | 51.8 | (36,65) | 48.6 | (33,77) | 47.8 | (35,67) | 51.1 | (30,62) |
| **WT monovalent Dose 1 and 2** | Pfizer | 53 | 88.3% | 14 | 87.5% | 17 | 89.5% | 14 | 93.3% | 8 | 80.0% |
| | Moderna | 6 | 10.0% | 2 | 12.5% | 2 | 10.5% | 1 | 6.7% | 1 | 10.0% |
| | Janssen | 1 | 1.7% | - | - | - | - | - | - | 1 | 10.0% |
| **WT monovalent Dose 3** | Pfizer | 51 | 85.0% | 14 | 87.5% | 16 | 84.2% | 14 | 93.3% | 7 | 70.0% |
| | Moderna | 9 | 15.0% | 2 | 12.5% | 3 | 15.8% | 1 | 6.7% | 3 | 30.0% |
| **WT monovalent Dose 4** | Pfizer | 18 | 30.0% | 5 | 31.3% | 6 | 31.6% | 3 | 20.0% | 4 | 40.0% |
| | Moderna | 8 | 13.3% | 4 | 25.0% | 2 | 10.5% | - | - | 2 | 20.0% |
| | None | 34 | 56.7% | 7 | 43.8% | 11 | 57.9% | 12 | 80.0% | 4 | 40.0% |
| **BA.5 bivalent booster** | Pfizer | 38 | 63.3% | 9 | 56.3% | 16 | 84.2% | 8 | 53.3% | 5 | 50.0% |
| | Moderna | 22 | 36.7% | 7 | 43.8% | 3 | 15.8% | 7 | 46.7% | 5 | 50.0% |
| **XBB.1.5 monovalent booster** | Pfizer | 20 | 33.3% | 8 | 50.0% | - | - | 7 | 46.7% | 5 | 50.0% |
| | Moderna | 21 | 35.0% | 8 | 50.0% | - | - | 8 | 53.3% | 5 | 50.0% |
| | None | 19 | 31.7% | - | - | 19 | 100.0% | - | - | - | - |
| | **Sera Days Pre XBB** | 26.5 | (1,74) | 19.8 | (1,74) | 30.8 | (3,69) | 28.1 | (2,69) | 26.8 | (7,55) |
| | **Sera Days Post XBB** | 26.4 | (20,34) | 26.0 | (21,32) | 27.8 | (22,30) | 25.9 | (20,34) | 24.9 | (21,30) |

**Table S2. Participant details, related to Figure 2.** Details are listed for each participant including demographic, vaccine, infection, and sera collection information.

| Sample ID | Age | Gender | Infection period | Vaccine type | | | | | | Days pre/post vaccine/infection | | Intervals between 1st dose to | | | | | |
|---|---|---|---|---|---|---|---|---|---|---|---|---|---|---|---|---|---|
| | | | | WT monovalent vaccine | | | | BA.5 bivalent booster | XBB.1.5 Monovalent booster | | | | | | | | |
| | | | | dose 1 | dose 2 | dose 3 | dose 4 | | | pre sample | post sample | 2nd dose | 3rd dose | 4th dose | BA.5 bivalent booster | Omicron infection | XBB.1.5 Monovalent booster |
| XBB.1.5 MV (n=16) | | | | | | | | | | | | | | | | | |
| 1 | 62 | Female | - | Pfizer | Pfizer | Pfizer | Pfizer | Pfizer | Moderna | 21 | 27 | 22 | 268 | 491 | 657 | - | 993 |
| 2 | 59 | Male | - | Pfizer | Pfizer | Pfizer | Pfizer | Pfizer | Pfizer | 6 | 23 | 21 | 269 | 491 | 622 | - | 1001 |
| 3 | 65 | Female | - | Pfizer | Pfizer | Pfizer | Pfizer | Pfizer | Pfizer | 24 | 28 | 22 | 266 | 503 | 637 | - | 990 |
| 4 | 59 | Female | - | Pfizer | Pfizer | Pfizer | - | Pfizer | Pfizer | 9 | 28 | 21 | 289 | - | 624 | - | 992 |
| 5 | 55 | Female | - | Pfizer | Pfizer | Pfizer | Moderna | Moderna | Moderna | 32 | 25 | 21 | 272 | 472 | 637 | - | 995 |
| 6 | 38 | Male | - | Moderna | Moderna | Moderna | - | Moderna | Pfizer | 22 | 27 | 28 | 235 | - | 539 | - | 914 |
| 7 | 64 | Female | - | Pfizer | Pfizer | Pfizer | Pfizer | Pfizer | Pfizer | 16 | 32 | 21 | 270 | 455 | 606 | - | 976 |
| 8 | 55 | Female | - | Pfizer | Pfizer | Pfizer | Moderna | Moderna | Moderna | 23 | 26 | 21 | 291 | 487 | 648 | - | 1005 |
| 9 | 56 | Female | - | Pfizer | Pfizer | Pfizer | - | Pfizer | Pfizer | 26 | 21 | 21 | 377 | - | 634 | - | 993 |
| 10 | 40 | Male | - | Pfizer | Pfizer | Pfizer | - | Moderna | Moderna | 3 | 22 | 21 | 268 | - | 584 | - | 969 |
| 11 | 50 | Male | - | Pfizer | Pfizer | Pfizer | Moderna | Pfizer | Moderna | 8 | 31 | 21 | 235 | 444 | 621 | - | 996 |
| 12 | 54 | Female | - | Pfizer | Pfizer | Pfizer | Moderna | Pfizer | Moderna | 1 | 22 | 21 | 329 | 490 | 623 | - | 990 |
| 13 | 38 | Female | - | Pfizer | Pfizer | Pfizer | - | Moderna | Pfizer | 74 | 30 | 21 | 276 | - | 619 | - | 985 |
| 14 | 56 | Female | - | Pfizer | Pfizer | Pfizer | Pfizer | Pfizer | Moderna | 10 | 22 | 21 | 287 | 507 | 654 | - | 1016 |
| 15 | 36 | Male | - | Moderna | Moderna | Moderna | - | Moderna | Moderna | 26 | 26 | 27 | 232 | - | 526 | - | 905 |
| 16 | 42 | Female | - | Pfizer | Pfizer | Pfizer | - | Moderna | Pfizer | 16 | 26 | 21 | 239 | - | 520 | - | 910 |
| XBB infx (n=19) | | | | | | | | | | | | | | | | | |
| 1 | 33 | Female | 2023.02 | Pfizer | Pfizer | Pfizer | | Pfizer | - | 5 | 30 | 21 | 334 | - | 643 | 151 | - |
| 2 | 38 | Female | 2023.08 | Pfizer | Pfizer | Pfizer | - | Pfizer | - | 53 | 27 | 24 | 294 | - | 666 | 307 | - |
| 3 | 59 | Female | 2023.08 | Pfizer | Pfizer | Pfizer | Pfizer | Pfizer | - | 37 | 26 | 21 | 295 | 472 | 660 | 280 | - |
| 4 | 44 | Male | 2023.05 | Pfizer | Pfizer | Pfizer | Pfizer | Moderna | - | 69 | 30 | 21 | 243 | 447 | 621 | 260 | - |
| 5 | 54 | Female | 2023.09 | Pfizer | Pfizer | Pfizer | Pfizer | Pfizer | - | 37 | 28 | 21 | 222 | 421 | 602 | 306 | - |
| 6 | 77 | Male | 2023.09 | Pfizer | Pfizer | Pfizer | Pfizer | Pfizer | - | 48 | 22 | 21 | 289 | 470 | 615 | 365 | - |
| 7 | 38 | Female | 2023.03 | Pfizer | Pfizer | Moderna | - | Pfizer | - | 14 | 28 | 21 | 278 | - | 613 | 99 | - |
| 8 | 59 | Female | 2023.05 | Pfizer | Pfizer | Pfizer | Pfizer | Pfizer | - | 24 | 30 | 25 | 281 | 469 | 613 | 249 | - |
| 9 | 41 | Female | 2023.02 | Pfizer | Pfizer | Pfizer | - | Pfizer | - | 24 | 29 | 21 | 289 | - | 659 | 108 | - |
| 10 | 38 | Female | 2023.08 | Pfizer | Pfizer | Pfizer | - | Pfizer | - | 3 | 30 | 21 | 291 | - | 660 | 305 | - |
| 11 | 42 | Male | 2023.04 | Pfizer | Pfizer | Pfizer | - | Moderna | - | 24 | 28 | 21 | 269 | - | 615 | 229 | - |
| 12 | 38 | Female | 2023.08 | Pfizer | Pfizer | Pfizer | - | Pfizer | - | 16 | 24 | 21 | 304 | - | 644 | 325 | - |
| 13 | 56 | Female | 2023.09 | Pfizer | Pfizer | Pfizer | Pfizer | Pfizer | - | 54 | 29 | 21 | 290 | 492 | 645 | 342 | - |
| 14 | 53 | Female | 2023.02 | Pfizer | Pfizer | Pfizer | - | Pfizer | - | 16 | 27 | 22 | 320 | - | 645 | 140 | - |
| 15 | 40 | Female | 2023.05 | Pfizer | Pfizer | Pfizer | - | Pfizer | - | 21 | 28 | 21 | 279 | - | 658 | 194 | - |
| 16 | 49 | Female | 2023.07 | Pfizer | Pfizer | Pfizer | - | Moderna | - | 63 | 30 | 22 | 328 | - | 641 | 264 | - |
| 17 | 59 | Female | 2023.04 | Moderna | Moderna | Moderna | Moderna | Moderna | - | 25 | 24 | 28 | 268 | 505 | 584 | 174 | - |
| 18 | 51 | Female | 2023.05 | Moderna | Moderna | Moderna | Moderna | Moderna | - | 39 | 29 | 28 | 253 | 441 | 554 | 228 | - |
| 19 | 54 | Female | 2023.08 | Pfizer | Pfizer | Pfizer | | Pfizer | - | 13 | 29 | 23 | 284 | - | 632 | 330 | - |
| Omicron infex + XBB.1.5 MV (n=25) | | | | | | | | | | | | | | | | | |
| subgroup 1: pre-XBB Omicron infx + XBB.1.5 MV (n=15) | | | | | | | | | | | | | | | | | |
| 1 | 41 | Female | 2022.09 | Moderna | Moderna | Moderna | - | Pfizer | Moderna | 57 | 25 | 28 | 330 | - | 715 | 624 | 1009 |
| 2 | 61 | Female | 2022.04 | Pfizer | Pfizer | Pfizer | - | Moderna | Moderna | 22 | 34 | 22 | 302 | - | 649 | 487 | 1006 |
| 3 | 53 | Female | 2022.04 | Pfizer | Pfizer | Pfizer | - | Pfizer | Moderna | 35 | 25 | 21 | 275 | - | 626 | 471 | 999 |
| 4 | 49 | Male | 2022.01 | Pfizer | Pfizer | Pfizer | - | Pfizer | Pfizer | 12 | 22 | 21 | 265 | - | 691 | 320 | 956 |
| 5 | 67 | Female | 2022.07 | Pfizer | Pfizer | Pfizer | Pfizer | Moderna | Moderna | 42 | 29 | 21 | 271 | 473 | 690 | 569 | 992 |
| 6 | 52 | Female | 2022.04 | Pfizer | Pfizer | Pfizer | Pfizer | Pfizer | Pfizer | 27 | 25 | 21 | 266 | 436 | 599 | 460 | 973 |
| 7 | 48 | Female | 2022.01 | Pfizer | Pfizer | Pfizer | - | Moderna | Pfizer | 47 | 22 | 21 | 254 | - | 554 | 291 | 925 |
| 8 | 43 | Male | 2022.01 | Pfizer | Pfizer | Pfizer | - | Pfizer | Pfizer | 29 | 20 | 21 | 265 | - | 614 | 359 | 977 |
| 9 | 53 | Female | 2022.10 | Pfizer | Pfizer | Pfizer | - | Pfizer | Pfizer | 21 | 25 | 21 | 279 | - | 760 | 638 | 997 |
| 10 | 40 | Female | 2022.04 | Pfizer | Pfizer | Pfizer | - | Moderna | Moderna | 69 | 21 | 24 | 276 | - | 638 | 467 | 1003 |
| 11 | 37 | Female | 2022.05 | Pfizer | Pfizer | Pfizer | - | Moderna | Moderna | 8 | 20 | 21 | 280 | - | 643 | 508 | 1009 |
| 12 | 43 | Female | 2022.01 | Pfizer | Pfizer | Pfizer | - | Moderna | Pfizer | 3 | 32 | 21 | 234 | - | 566 | 309 | 913 |
| 13 | 36 | Male | 2022.08 | Pfizer | Pfizer | Pfizer | - | Moderna | Moderna | 2 | 30 | 21 | 316 | - | 655 | 583 | 980 |
| 14 | 59 | Female | 2022.04 | Pfizer | Pfizer | Pfizer | Pfizer | Pfizer | Moderna | 6 | 29 | 21 | 277 | 543 | 610 | 448 | 986 |
| 15 | 35 | Female | 2022.06 | Pfizer | Pfizer | Pfizer | Pfizer | Pfizer | Pfizer | 42 | 29 | 22 | 277 | - | 651 | 527 | 1008 |
| subgroup 2: XBB infx + XBB.1.5 MV (n=10) | | | | | | | | | | | | | | | | | |
| 1 | 54 | Female | 2023.02 | Pfizer | Pfizer | Pfizer | Moderna | Pfizer | Moderna | 21 | 22 | 21 | 262 | 492 | 632 | 774 | 1003 |
| 2 | 62 | Female | 2023.04 | Pfizer | Pfizer | Pfizer | Pfizer | Pfizer | Moderna | 40 | 29 | 21 | 294 | 516 | 630 | 845 | 1000 |
| 3 | 61 | Female | 2023.04 | Pfizer | Pfizer | Pfizer | Pfizer | Moderna | Moderna | 53 | 26 | 21 | 275 | 519 | 625 | 843 | 1004 |
| 4 | 30 | Female | 2023.08 | Pfizer | Pfizer | Pfizer | - | Moderna | Pfizer | 21 | 22 | 21 | 218 | - | 580 | 922 | 880 |
| 5 | 58 | Female | 2023.02 | Pfizer | Pfizer | Pfizer | - | Pfizer | Pfizer | 10 | 22 | 21 | 284 | - | 682 | 1000 | 776 |
| 6 | 61 | Male | 2023.05 | Pfizer | Pfizer | Moderna | - | Moderna | Moderna | 7 | 30 | 21 | 231 | - | 541 | 917 | 800 |
| 7 | 42 | Female | 2023.03 | Pfizer | Pfizer | Pfizer | Pfizer | Moderna | Pfizer | 13 | 29 | 21 | 266 | 477 | 608 | 800 | 996 |
| 8 | 62 | Female | 2023.06 | Pfizer | Pfizer | Pfizer | Pfizer | Pfizer | Pfizer | 18 | 21 | 21 | 277 | 439 | 643 | 895 | 1002 |
| 9 | 46 | Male | 2023.05 | J&J-Janssen | J&J-Janssen | Moderna | - | Pfizer | Pfizer | 55 | 24 | 214 | 386 | - | 543 | 776 | 921 |
| 10 | 35 | Female | 2023.06 | Moderna | Moderna | Moderna | Moderna | Moderna | Moderna | 30 | 24 | 28 | 342 | 437 | 616 | 892 | 1014 |

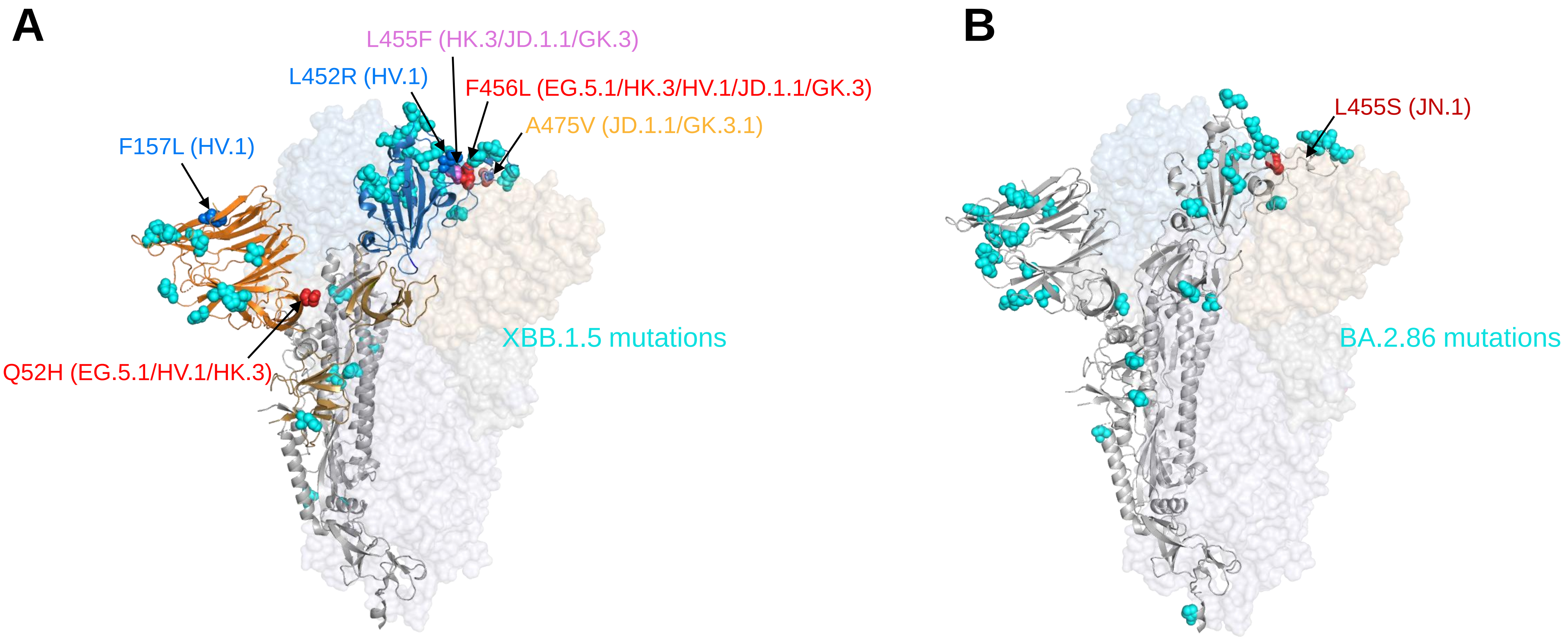


**Figure S1. Spike mutations found in emerging SARS-CoV-2 Omicron subvariants, related to Figure 1.**
A. Mutations found in EG.5.1, HV.1, HK.3, JD.1.1, and GK.3 on top of the XBB.1.5 spike.
B. Location of the L455S mutation in JN.1 on top of the BA.2.86 spike.
Mutations present in XBB.1.5 and BA.2.86 are highlighted in cyan. The spike protein structure is obtained under PDB ID: 6ZGE.

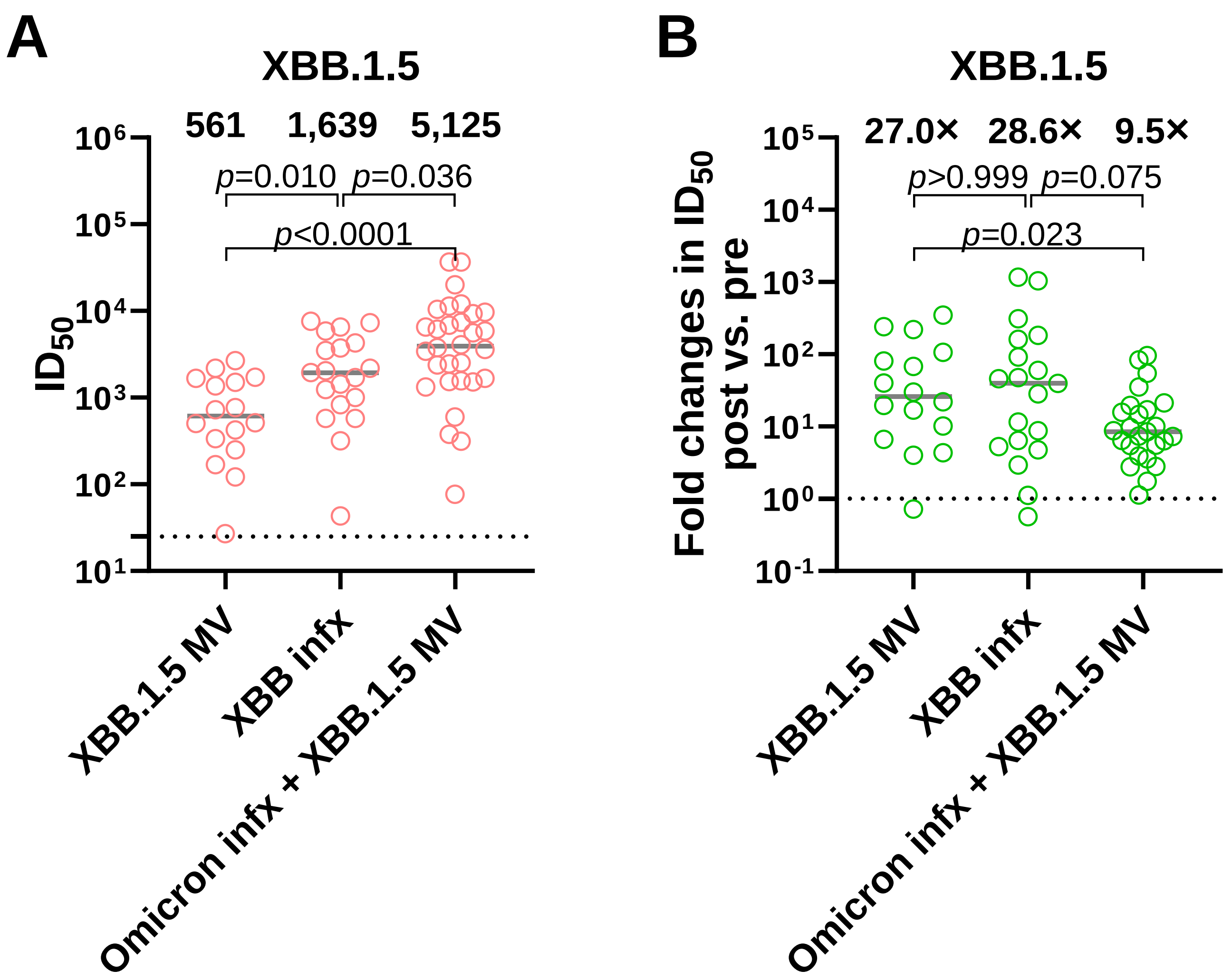


**Figure S2. Neutralizing antibody titers against XBB.1.5 induced by XBB.1.5 monovalent vaccine, XBB infection or Omicron infection + XBB.1.5 monovalent vaccine. Related to Figure 2.**

Serum neutralizing $ID_{50}$ titers against XBB.1.5 of the indicated cohorts post vaccination or infection.

Fold increases in neutralizing $ID_{50}$ titers of the indicated three cohorts against XBB.1.5 between pre and post vaccination or infection.

Geometric mean $ID_{50}$ titers (**A**) and geometric mean fold changes (**B**) are shown above *p* values. Comparisons were made by Mann-Whitney unpaired *t* tests. The dotted lines represent the assay limit of detection of 25 (**A**) and fold change of 1 (**B**).

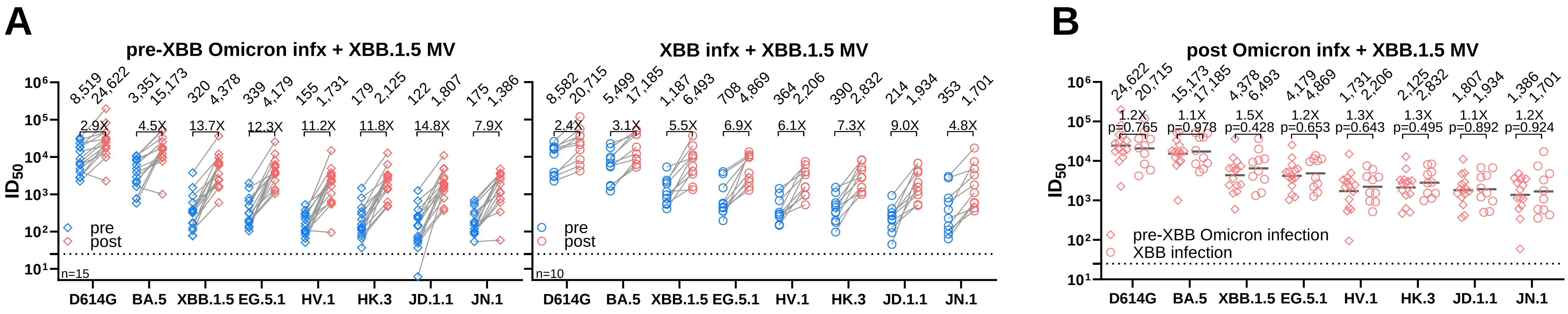


**Figure S3. Neutralizing antibody titers of two subgroups of the "Omicron infx + XBB.1.5 MV" cohort, related to Figure 2.**

A. Comparison in neutralizing antibody titers before and after an XBB.1.5 mRNA vaccine booster following a pre-XBB Omicron infection or an XBB infection. Geometric mean $ID_{50}$ titers are shown along with the fold change between pre and post XBB.1.5 vaccination against each indicated virus.

B. Comparison in neutralizing antibody titers after an XBB.1.5 mRNA vaccine booster following a pre-XBB Omicron infection or an XBB infection. Statistical analyses were made by Mann-Whitney unpaired *t* tests. Geometric mean $ID_{50}$ titers are shown along with the fold changes between the two subgroups.

Participants from the "Omicron infx + XBB.1.5 MV" cohort were stratified into two groups based on the infection strain. The dotted line represents the assay limit of detection of 25. "n" denotes the sample size.

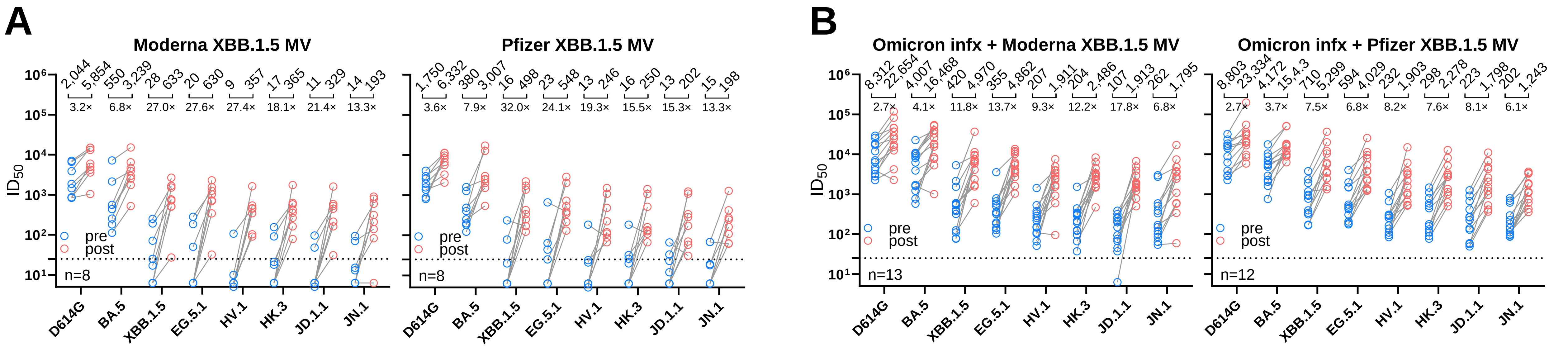


**Figure S4. Neutralizing antibody titers before and after a Moderna or Pfizer XBB.1.5 mRNA vaccine booster, related to Figure 2.** Participants from the "XBB.1.5 MV" cohort (**A**) and "Omicron infx + XBB.1.5 MV" (**B**) were stratified into two groups based on the vaccine manufacturer. Geometric mean $ID_{50}$ titers are shown along with the fold change between pre and post XBB.1.5 vaccination against each indicated virus. The dotted line represents the assay limit of detection of 25. "n" denotes the sample size.
